# Membrane affinity difference between MinD monomer and dimer is not crucial for MinD gradient formation in *Bacillus subtilis*

**DOI:** 10.7554/eLife.101520

**Published:** 2026-09-02

**Authors:** Laura C Bohorquez, Henrik Strahl, Davide Marenduzzo, Martin J Thiele, Frank Burmann, Leendert Hamoen

**Affiliations:** 1 Swammerdam Institute for Life Sciences, University of Amsterdam Amsterdam Netherlands; 2 Centre for Bacterial Cell Biology, Biosciences Institute, Newcastle University Newcastle upon Tyne United Kingdom; 3 SUPA, School of Physics and Astronomy, The University of Edinburgh Edinburgh United Kingdom; Washington University in St. Louis United States; University of Geneva Switzerland

**Keywords:** MinD, membrane affinity, protein gradient, *B. subtilis*

## Abstract

Proteins can diffuse micrometers in seconds, yet bacterial cells are able to maintain stable protein gradients. The best-studied bacterial protein gradient is the Min system of *Escherichia coli*. In rod-shaped bacteria, the MinCD proteins prevent formation of minicells by inhibiting FtsZ polymerization close to the cell poles. In *E. coli*, these proteins oscillate between cell poles within a minute, resulting in an increased MinCD concentration at the poles. This oscillation is caused by the interaction between MinD and the protein MinE, which form an ATP-driven reaction-diffusion system, whereby the ATPase MinD cycles between a monomeric cytosolic and a dimeric membrane-attached state. *Bacillus subtilis* also has MinCD, but lacks MinE. In this case, MinCD forms a static gradient that requires the transmembrane protein MinJ, located at cell poles and cell division sites. A recent reaction-diffusion model was successful in recreating the MinD gradient in *B. subtilis*, assuming that MinD cycles between cytosol and membrane, like in *E. coli*. Here, we show that the monomeric and dimeric states of *B. subtilis* MinD have comparable membrane affinities, that MinD interacts with MinJ as a dimer, and that MinJ is not required for membrane localization of MinD. Based on these new findings, we tested different models, using kinetic Monte Carlo simulations, and found that a difference in diffusion rate between the monomer and dimer, rather than a difference in membrane affinity, is important for *B. subtilis* MinCD gradient formation.

## Introduction

Bacterial cells are capable of positioning proteins at midcell and cell poles, and can form protein gradients without the support of specific membrane compartments or guidance by cytoskeleton elements, as found in eukaryotic cells. A well-known example is the formation of a MinCD protein gradient along the cell axis of rod-shaped *E. coli* and *B. subtilis* cells. These proteins ensure that cell division occurs at midcell and not close to cell poles (Ramm et al., 2019). The formation of these protein gradients is remarkable considering that the diffusion of proteins in cells is in the order of μm squared per second, whereas these bacteria are only a few µm in length (Kumar et al., 2010).

The Min system of *E. coli* is the best-studied gradient system and comprises the membrane-associated MinD and MinE proteins that form a reaction-diffusion couple (Howard et al., 2001; Meinhardt and de Boer, 2001; Huang et al., 2003), resulting in a MinCD gradient that oscillates between cell poles in a matter of seconds (Hu and Lutkenhaus, 1999; Raskin and de Boer, 1999). This system has been reconstructed on artificial membranes resulting in dynamic wave patterns (Loose et al., 2008) and extensively simulated to understand the reaction parameters (e.g. Howard et al., 2001; Meinhardt and de Boer, 2001; Huang et al., 2003; Kruse, 2002; Di Ventura and Sourjik, 2011; Würthner et al., 2022; Glock et al., 2019). *B. subtilis* also forms a MinD gradient that decreases in concentration from cell poles and nascent cell division sites toward the middle of the cell. However, unlike *E. coli*, this gradient does not oscillate (Marston et al., 1998; Marston and Errington, 1999; Gregory et al., 2008). In this study, we investigated how the MinCD gradient is formed in *B. subtilis*.

The core of the Min system consists of the protein couple MinC and MinD that prevent aberrant polymerization of the key cell division protein FtsZ close to newly formed septa or cell poles (Gregory et al., 2008; Levin et al., 1998; Bi and Lutkenhaus, 1993). MinC inhibits polymerization of FtsZ by direct protein-protein interactions and needs to bind to the Walker A-type ATPase MinD for its recruitment to the polar regions of the cell (de Boer et al., 1989; de Boer et al., 1991; Hu and Lutkenhaus, 2000; Cordell et al., 2001). Binding of ATP leads to MinD dimerization (Park et al., 2012; Hu and Lutkenhaus, 2003) and subsequent association with the cell membrane, whereby its C-terminal amphipathic helix functions as a membrane anchor (Hu and Lutkenhaus, 2003; Szeto et al., 2003). MinC is recruited to the cell membrane by association with MinD dimers (Hu and Lutkenhaus, 2003; Hu et al., 2003; de Boer et al., 1992). The *E. coli* Min system comprises a third protein, the peripheral membrane protein MinE, that interacts with MinD, thereby displacing MinC and stimulating the ATPase activity of MinD, which ultimately triggers the release of MinD from the membrane (Hu et al., 2003; Park et al., 2017; Hu and Lutkenhaus, 2001; Hu et al., 2002; Lackner et al., 2003). The interaction with MinD causes a conformational change in MinE that stimulates its membrane affinity (Park et al., 2017). These reciprocal interactions of MinD and MinE represent a natural reaction-diffusion system, resulting in the oscillation of MinCD proteins between cell poles with a periodicity of approximately 50 s (Hu and Lutkenhaus, 1999; Raskin and de Boer, 1999; Loose et al., 2008). Through this oscillation, the average concentration of MinC is higher near the cell poles and lower at midcell, thereby inhibiting polymerization of FtsZ close to cell poles and favoring Z-ring formation at midcell (Rowlett and Margolin, 2013).

The MinCD proteins of *E. coli* and *B. subtilis* are highly conserved. However, *B. subtilis* does not encode a MinE homologue and instead requires the proteins DivIVA and MinJ for the proper polar and septal localization of MinCD (Bramkamp et al., 2008; Patrick and Kearns, 2008). DivIVA is a general scaffold protein conserved in Gram-positive bacteria that associates with strong negatively curved membrane areas at the base of division septa, resulting in polarly located DivIVA clusters after division is completed (Lenarcic et al., 2009; Ramamurthi and Losick, 2009). The integral membrane protein MinJ interacts with DivIVA and shows the same localization pattern. MinJ also interacts with MinD, and inactivation of MinJ eliminates a MinCD gradient, resulting in filamentous cells and minicell formation due to uncontrolled activity of MinC (Bramkamp et al., 2008; Patrick and Kearns, 2008). Since DivIVA is recruited to midcell in the early stages of septum synthesis when a strong concave membrane area is formed (Lenarcic et al., 2009), the Min proteins are already present when septum synthesis is ongoing, and their primary activity is to inhibit Z-ring formation next to nascent division sites (Gregory et al., 2008).

The ATP-driven cycle between membrane association and dissociation of MinD is critical to the formation of the oscillating MinD gradient in *E. coli*. Mathematical modeling has shown that such an ATP-driven reversible membrane attachment cycle can also explain the formation of a MinD gradient in *B. subtilis* (Howard, 2004; Feddersen et al., 2021). However, the C-terminal amphipathic alpha helix of *B. subtilis* MinD has a much stronger affinity for the cell membrane compared to its *E. coli* counterpart, and when fused to GFP can recruit this protein to the cell membrane, which the *E. coli* MinD C-terminal membrane anchor is not capable of Szeto et al., 2003. In fact, a MinD point mutant that prevents binding of ATP and maintains the protein in its monomeric state is still recruited to the *B. subtilis* cell membrane (Karoui and Errington, 2001). It is therefore unclear whether dimerization-dependent changes in the membrane affinity of MinD are crucial for the formation of a *B. subtilis* MinCD gradient.

In this study, we analyzed and manipulated the membrane affinity of *B. subtilis* MinD, and combined in vivo experiments with in silico Monte Carlo simulations to assess the role of membrane affinity in the formation of a MinCD gradient. The main difference between our kinetic Monte Carlo model and previous mathematical models based on reaction-diffusion equations is that our treatment is particle-based, rather than continuum field-based, and naturally includes the effect of stochastic fluctuations, i.e., noise of the system. The experiments demonstrated that, unlike in *E. coli*, a difference in membrane affinity between MinD monomer and dimer is not critical for the creation of a MinCD gradient. In addition, we examined how differences in MinD membrane affinity affect the recruitment of MinC to the membrane, a process that is critical for the FtsZ-regulating activity of the Min system.

## Results

### Effect of dimerization on the localization of *B. subtilis* MinD

**Figure 1.**
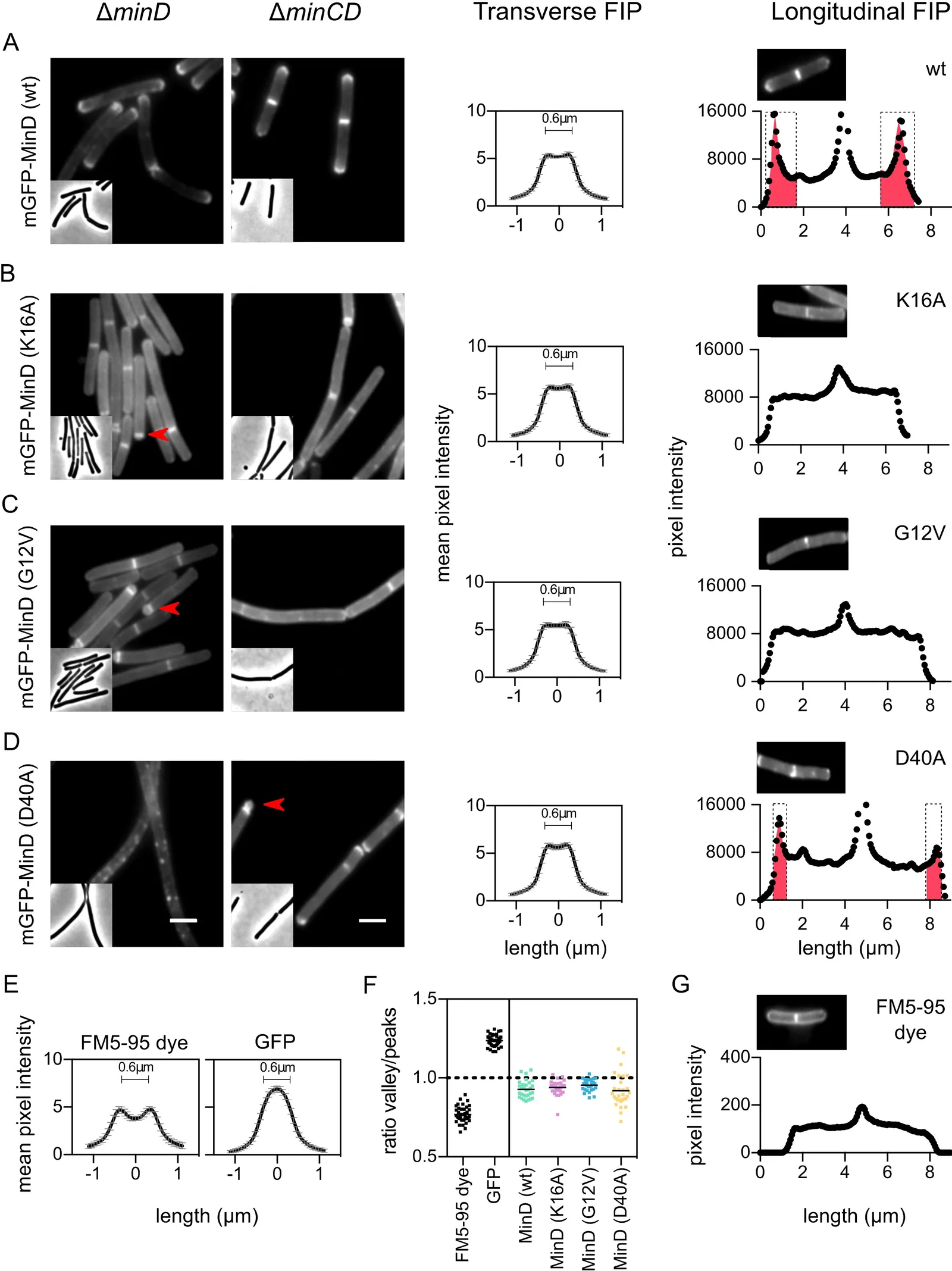
Localization of monomeric and dimeric MinD variants. Cellular localization of (**A**) wild-type MinD, (**B**) MinD K16A, (**C**) MinD G12V, and (**D**) MinD D40A. Localization was monitored by an N-terminal monomeric GFP (mGFP) fusion. Fusion proteins were expressed in either a Δ*minD* or Δ*minCD* background. Fluorescence images (left panels) and corresponding phase contrast images (inset) are shown in the left panels. Some minicells are indicated with red arrows. Scale bar is 2 μm. Middle panels show the transverse fluorescence intensity profiles (FIPs) with standard deviations calculated using an average of at least 30 cells (Δ*minCD* background) per dataset. Right panels depict the longitudinal FIPs using the Δ*minCD* background. The manually shaded red areas highlight the polar gradients. Additional examples for wild-type MinD and the D40A variants are shown in Figure 1—figure supplement 1. (**E**) Transversal FIP with standard deviations of exponentially growing wild-type cells stained with fluorescence membrane dye FM5-95, and wild-type cells expressing GFP are shown as controls. (**F**) Membrane affinities, with median values, estimated from the valley/peak ratios shown in the middle panels of (**A–D**) and controls (**E**). (**G**) Longitudinal FIP along the exponentially growing wild-type cells stained with the fluorescence membrane dye FM5-95. Strains used in (**A**) LB249 and LB305, (**B**) LB250 and LB306, (**C**) LB251 and LB307, (**D**) LB252 and LB308, (**E**) LB609 and (**G**) 168. Figure 1—source data 1.Fluorescence intensity data Figure 1.

We began by examining how much the membrane affinity of monomeric *B. subtilis* MinD differs from the dimeric form by constructing MinD variants that are fixed in one of these conformations. Dimerization of MinD and other members of the MinD/ParA protein family requires binding of ATP. The conserved lysine in the Walker A-type ATPase domain, amino acid position 16 in the protein (Appendix 1—figure 1), forms hydrogen bonds with the phosphate groups of the nucleotide, as revealed in the crystal structure of *Pyrococcus furiosus* MinD (Hayashi et al., 2001). Exchanging this residue into an alanine (K16A, apo monomer) prevents ATP binding and formation of dimers in several Walker motif-containing proteins, including *E. coli* MinD and RecA, and *B. subtilis* ParA/Soj (Wu et al., 2011; Story and Steitz, 1992; Leonard et al., 2005). The conserved glycine at position 12 makes contacts with the γ-phosphate of ATP across the dimer interface (Wu et al., 2011). It has been shown that changing this residue into a valine in *B. subtilis* ParA causes a steric clash in the active site (G12V, ATP-bound monomer), thereby preventing dimerization while retaining ATP binding (Wu et al., 2011; Leonard et al., 2005). These two monomer-forcing amino acid exchanges (K16A and G12V) were introduced into *B. subtilis* MinD, which was fused at the N-terminus with GFP to follow its localization in the cell. It has been shown that a GFP-MinD fusion retains its biological activity (Marston and Errington, 1999), and the fusion protein was expressed at levels that resulted in normal-sized cells and the absence of minicells (Figure 1A). To prevent possible localization artifacts due to the weak dimerization characteristics of GFP (Landgraf et al., 2012), we used a monomeric GFP (mGFP) variant. The different MinD fusion proteins were expressed from the ectopic *amyE* locus, under control of the xylose-inducible *Pxyl* promoter (Lewis and Marston, 1999), in a strain lacking wild-type *minD* (∆*minD*), as well as in a strain lacking *minD* and *minC* (∆*minCD*). Western blot analysis showed that the different MinD variants were stable and expressed at levels comparable to that of wild-type MinD (Appendix 1—figure 2). Induction of mGFP-MinD with 0.1% xylose reduced minicell formation in a ∆*minD* background to wild-type levels (from 14.7% to 0.07%, n>300). However, the mGFP-MinD-K16A and -G12V variants were unable to prevent the formation of minicells (11.2% and 9.5%, respectively, n>600).

**Figure 1—figure supplement 1.**
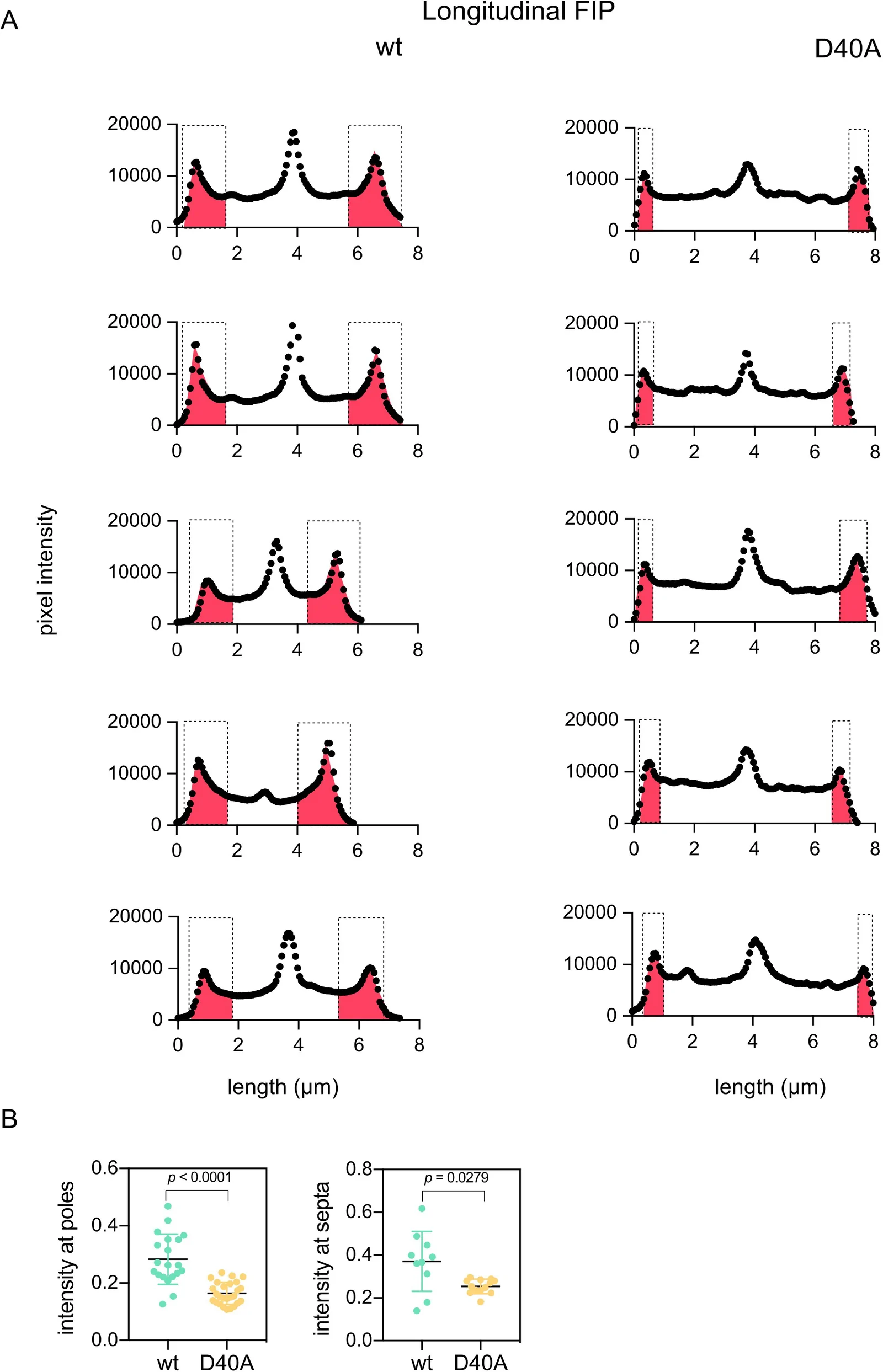
Longitudinal fluorescence intensity profiles (FIPs) of GFP-MinD wild-type and D40A variants. (**A**) Additional examples of longitudinal FIPs of GFP-MinD and GFP-MinD-D40A shown in Figure 1. Polar and septal areas were selected manually based on the fluorescence gradient. Polar areas are indicated in red. (**B**) Quantification of pixel intensities, with median values at poles and septa in the longitudinal FIP relative to the intensity of the total integrated intensity. Significance of difference was confirmed using a t-test. Figure 1—figure supplement 1—source data 1.Fluorescence intensity data Figure 1—figure supplement 1.

Figure 1B shows the effect of the K16A exchange on mGFP-MinD localization. In line with previous results (Karoui and Errington, 2001), preventing the binding of ATP abolishes the mGFP-MinD concentration gradient, which becomes clearer in longitudinal fluorescence intensity profiles (FIPs) (Figure 1, right panels). The fluorescence peaks visible at midcell are caused by the presence of two septal cell membrane layers, as indicated by the FIPs of a wild-type cell stained with the fluorescent membrane dye FM 5–95 (Figure 1G).

The monomeric ATP-binding G12V variant shows the same absence of a protein gradient as the K16A variant (Figure 1C). In contrast to *E. coli* MinD (Hu et al., 2002), both monomeric *B. subtilis* MinD mutants still bind to the cell membrane. To measure the membrane association in vivo, transverse FIPs were collected (Figure 1, middle panels), and the membrane affinities were estimated based on the ratio between valley (cytoplasmic) and peak (membrane) signals. This confirmed that the different monomeric mutants have a comparable membrane affinity as wild-type MinD (Figure 1F). As controls, we used the fluorescence membrane dye FM5-95 and cells expressing cytosolic GFP (Figure 1E).

It has been shown that exchanging a conserved aspartic acid residue in the ATPase domains of *B. subtilis* ParA and *E. coli* MinD blocks ATP hydrolysis and traps the proteins in a dimeric state (Wu et al., 2011; Leonard et al., 2005). When this aspartic acid residue, located at amino acid position 40 in the *B. subtilis* MinD sequence (Appendix 1—figure 1), was replaced by an alanine (D40A, ATP-bound dimer, ATP hydrolysis deficient) in the mGFP-MinD fusion, expression of this variant in a ∆*minD* strain resulted in highly filamentous cells with clusters of mGFP-MinD-D40A formed along the membrane (Figure 1D, left picture). Introduction of a *minC* deletion restored cell division (Figure 1D, right picture), indicating that the locked MinD dimer causes hyperactivation of MinC. Interestingly, in this ∆*minCD* background, the clusters disappeared and mGFP-MinD-D40A showed a clear polar and septal accumulation, although the polar and septal signal intensities were approximately 60–70% compared to that of wild-type cells (Figure 1—figure supplement 1), and the longitudinal protein gradient was clearly reduced (Figure 1D and Figure 1—figure supplement 1A). Quantification of the membrane affinity showed that the membrane affinity of the D40A variant is only slightly higher compared to the monomeric mutants (Figure 1F).

### Localization of trapped MinD dimer depends on MinJ

**Figure 2.**
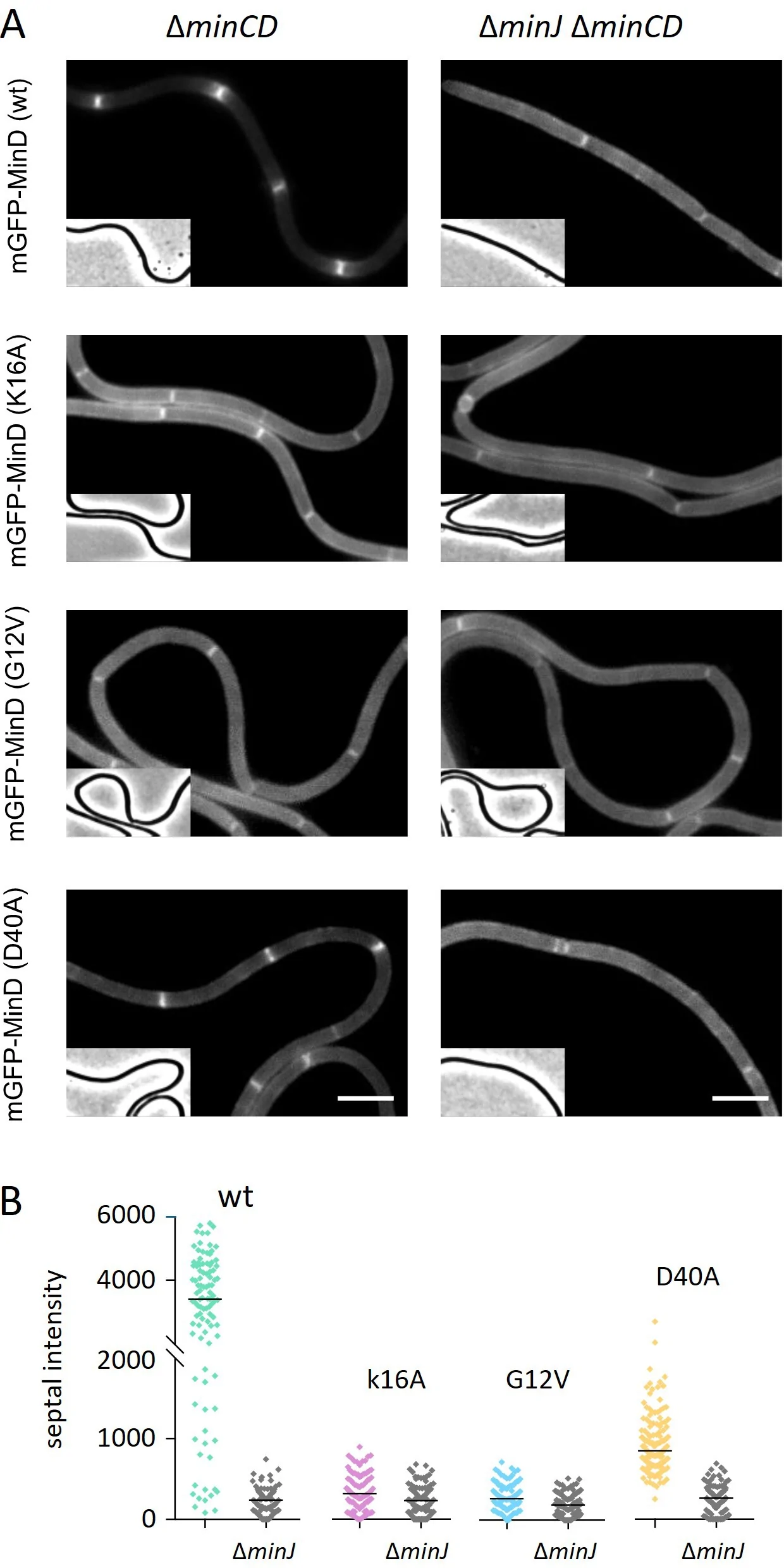
Effect of a *minJ* deletion on MinD-GFP localization. (**A**) Fluorescence microscopy images of Δ*minCD* and Δ*minJ*Δ*minCD* mutant cells expressing different monomeric GFP (mGFP)-MinD variants. Corresponding phase contrast images are shown in the insets. (**B**) Quantification of related septal fluorescence intensities, with median values at septa (n>100). Since the strongly filamentous Δ*minJ* strain is delicate to handle, cells were grown on agarose patches on microscopy slides. Scale bar is 5 μm. Strains used for wt: LB405 and LB409, K16A: LB406 and LB410, G12V: LB407 and LB411, and D40A: LB408 and LB412. Figure 2—source data 1.Fluorescence intensity data Figure 2.

The polar and septal localization of the D40A trapped dimer variant of MinD presumably relies on the interaction with MinJ. It has been reported that the absence of MinJ results in detachment of MinD from the membrane (Bramkamp et al., 2008; Feddersen et al., 2021). However, when we introduced the ∆*minJ* in our ∆*minCD* deletion strain background, we found that all MinD-GFP variants remained attached to the membrane, and the septal and polar localization of wild-type and the trapped dimer MinD disappeared (Figure 2). These results show that, while MinJ is not required for membrane attachment of MinD per se, it is required for polar and septal localization of MinD through interactions specifically with the dimeric form of MinD.

### Recruitment of MinC by the different MinD mutants

**Figure 3.**
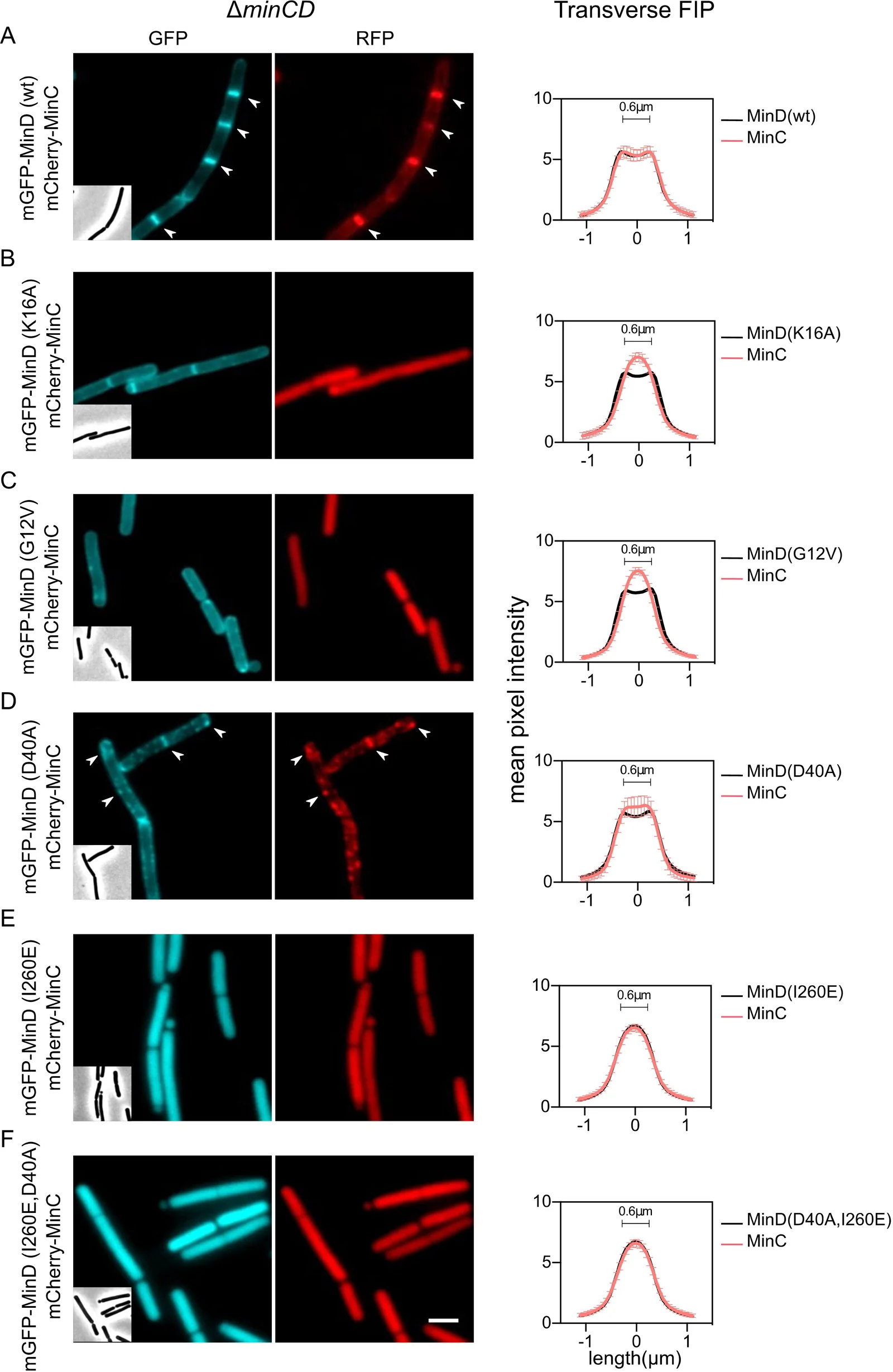
Membrane recruitment of MinC by MinD variants. Fluorescence microscopy images of cells expressing different monomeric GFP (mGFP)-MinD variants (cyan) and mCherry-MinC (red). Corresponding phase contrast images are shown in the insets. (**A**) Wild-type MinD, (**B**) MinD K16A, (**C**) MinD G12V, (**D**) MinD D40A, (**E**) MinD I260E, (**F**) MinD D40A, I260E. Right panels show the transverse fluorescence intensity profiles (FIPs) with standard deviations averaged over at least 30 cells. White arrows in (**D**) highlight colocalization. Scale bar is 2 μm. mGFP-MinD variants and mCherry-MinC were expressed in a Δ*minCD* background strain. Strains used: (**A**) LB318, (**B**) LB319, (**C**) LB320, (**D**) LB321, (**E**) LB643, and (**F**) LB644. Figure 3—source data 1.Fluorescence intensity data Figure 3.

Several biochemical studies with different bacterial species, including *B. subtilis*, have shown that MinC and MinD dimers can assemble into long polymers (Ghosal et al., 2014; Conti et al., 2015; Huang et al., 2018; Wang et al., 2022). However, the assembly of large MinCD multimers has not been observed in vivo, and a genetic study indicated that the formation of such polymers is not necessary for the function of the Min system in *E. coli* (Park et al., 2015). The D40A MinD variant showed a clustered membrane localization pattern that resolved into a smooth membrane pattern when *minC* was deleted (Figure 1D). This change in localization pattern might be related to the formation of higher-order MinCD assemblies. To confirm this, we introduced an active mCherry-MinC fusion to see whether mGFP-MinD and mCherry-MinC would colocalize when the former is fixed in a dimeric state. First, we examined the localization of MinC in the presence of monomeric K16A and G12V MinD variants. As shown in Figure 3B and C, the mCherry-MinC signal was dispersed throughout the cytosol in these strains, indicating a lack of interaction with monomeric MinD variants, in line with a previous report (Karoui and Errington, 2001). In the case of the mGFP-MinD-D40A variant, the mCherry-MinC expression was only induced for 1 hr to prevent excessive filamentation. As shown in Figure 3D, the mCherry-MinC signal showed foci along the membrane that often colocalized with mGFP-MinD-D40A foci, confirming that MinD dimerization is crucial for MinC interaction, and that both proteins can indeed form large assemblies in the cell, presumably in the form of MinCD copolymers.

The colocalization of the MinD-D40A and MinC fluorescent fusion proteins enabled us to investigate whether MinD dimerization is sufficient for MinC activity or whether localization at the cell membrane is also required. To test this, the membrane binding capacity of MinD was impaired by replacing isoleucine 260, located in the C-terminal membrane targeting amphipathic alpha helix, with a glutamate residue (Figure 3E; Strahl and Hamoen, 2010). Next, we introduced this mutation in the mGFP-MinD-D40A mutant to create a trapped MinD dimer that was no longer able to bind to the membrane. Expression of this cytosolic MinD variant did not result in cell filamentation (Figure 3F), indicating that interaction with MinD dimers alone is not sufficient to inhibit FtsZ polymerization. Rather, MinC needs to be localized and enriched to the membrane surface to be effective, which is not surprising since Z-ring formation occurs at the membrane periphery.

### Weak membrane interaction is important for MinD gradient formation

**Figure 4.**
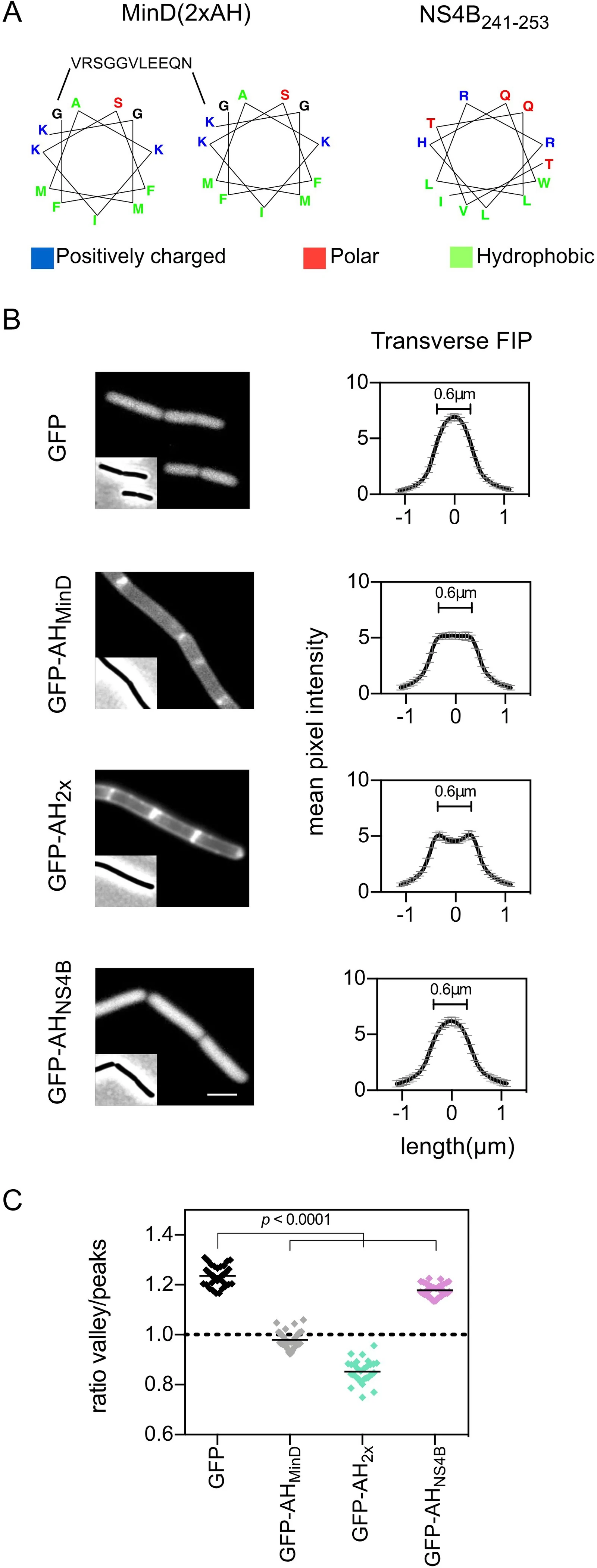
Membrane association of different amphipathic helices. (**A**) Schematic presentation of the tandem amphipathic helix and the weak amphipathic helix from Hepatitis C virus protein NS4B_241-253_. (**B**) Fluorescence microscopy images and transverse fluorescence intensity profiles (FIPs) with standard deviations of cells expressing the different amphipathic helix sequences fused to the C-terminus of GFP. A strain expressing cytoplasmic GFP was included for comparison. Scale bar is 2 μm. Strains used: FBB043 (GFP-AH_MinD_), FBB05 (GFP-AH_2x_), FBB046 (GFP-AH_NS4B_), LB609 (GFP). (**C**) Average membrane affinities calculated from the transverse FIPs (n>30). Significance of difference was confirmed using a t-test. Figure 4—source data 1.Fluorescence intensity data Figure 4.

The absence of a clear difference in membrane affinity between monomeric and dimeric MinD raised the question of whether reversible membrane binding is important for the formation of a protein gradient. To examine this, we tried to increase the membrane affinity of MinD by adding an extra copy of its C-terminal membrane targeting amphipathic helix. The extra amphipathic helix was connected to the original helix by a short 11 amino acid long flexible linker (Figure 4A). First, we tested whether such tandem amphipathic helix (2xAH) increases the membrane affinity by fusing it to mGFP. As shown in Figure 4, fusion of this tandem helix to mGFP increased the membrane association of the latter considerably when compared to a single MinD amphipathic helix.

**Figure 5.**
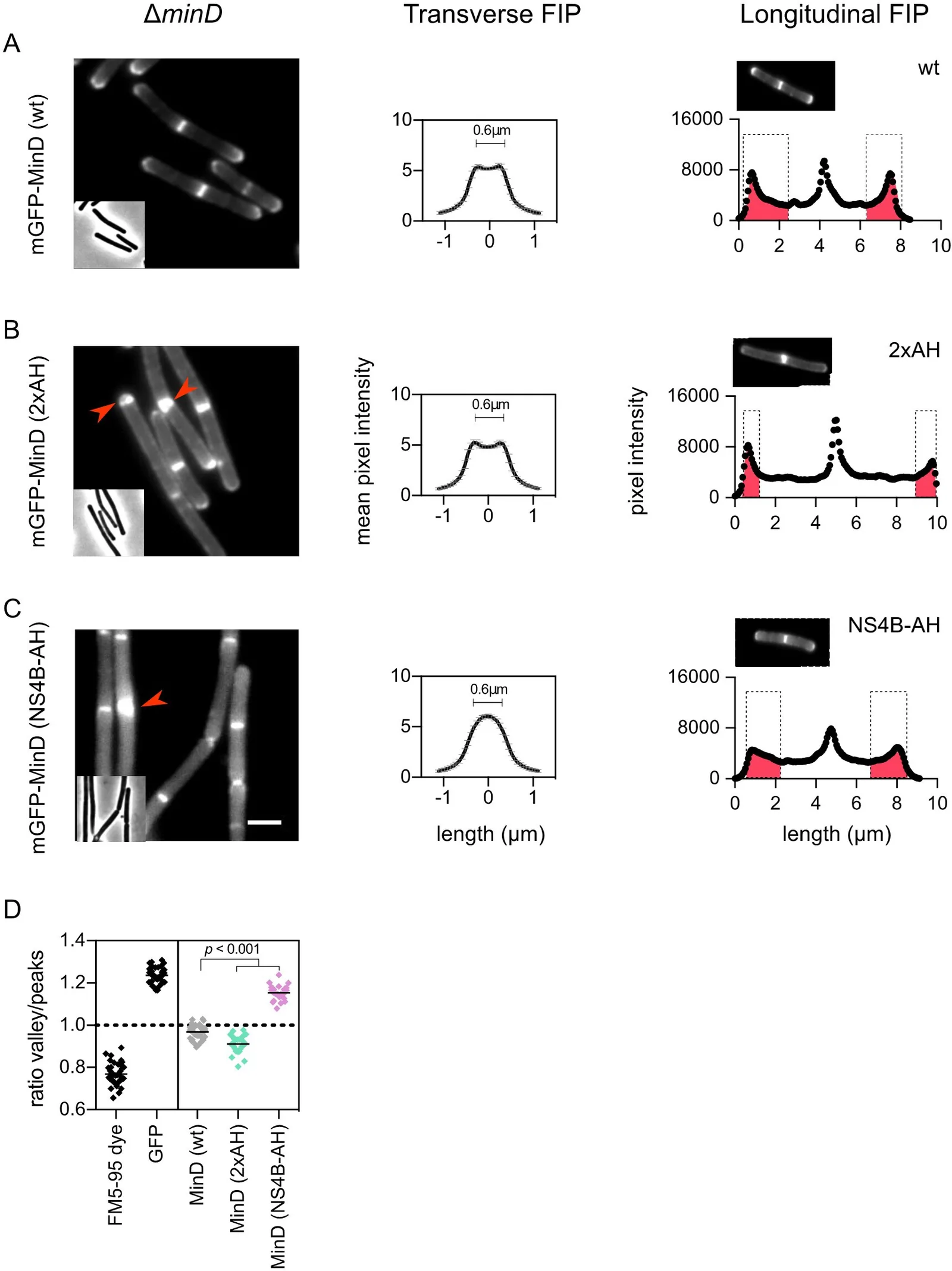
Membrane affinity affects MinD gradient. Fluorescence microscopy and fluorescence intensity profiles (FIPs) with standard deviations of cells expressing monomeric GFP (mGFP)-MinD containing either the native amphipathic helix (wt) (**A**), the tandem MinD amphipathic helix (2xAH) (**B**), or the weak Hepatitis C virus protein NS4B amphipathic helix (NS4B-AH) (**C**). The manually shaded red areas highlight the polar gradients. Additional examples are shown in Figure 5—figure supplement 1. (**D**) Relative membrane affinities with median values of the different mGFP-MinD variants were calculated from transverse FIPs (n>30). Significance of difference was confirmed using a t-test. The MinD variants were expressed in a Δ*minD* background. Phase contrast images are shown as insets. Some double septa are indicated with red arrows. Scale bar is 2 μm. Red areas in the intensity profiles highlight the polar gradients. Strains used in (**A**) LB249, (**B**) LB507, and (**C**) LB508. Figure 5—source data 1.Fluorescence intensity data Figure 5.

Next, we replaced the membrane targeting amphipathic helix of mGFP-MinD by the tandem amphipathic helix. Western blot analysis showed that this new C-terminus did not affect the protein levels to a significant degree (Appendix 1—figure 2). As shown in Figure 5B and D, the tandem helix notably increased the fluorescent membrane signal all along the cell membrane, thereby reducing the longitudinal fluorescence gradients.

**Figure 5—figure supplement 1.**
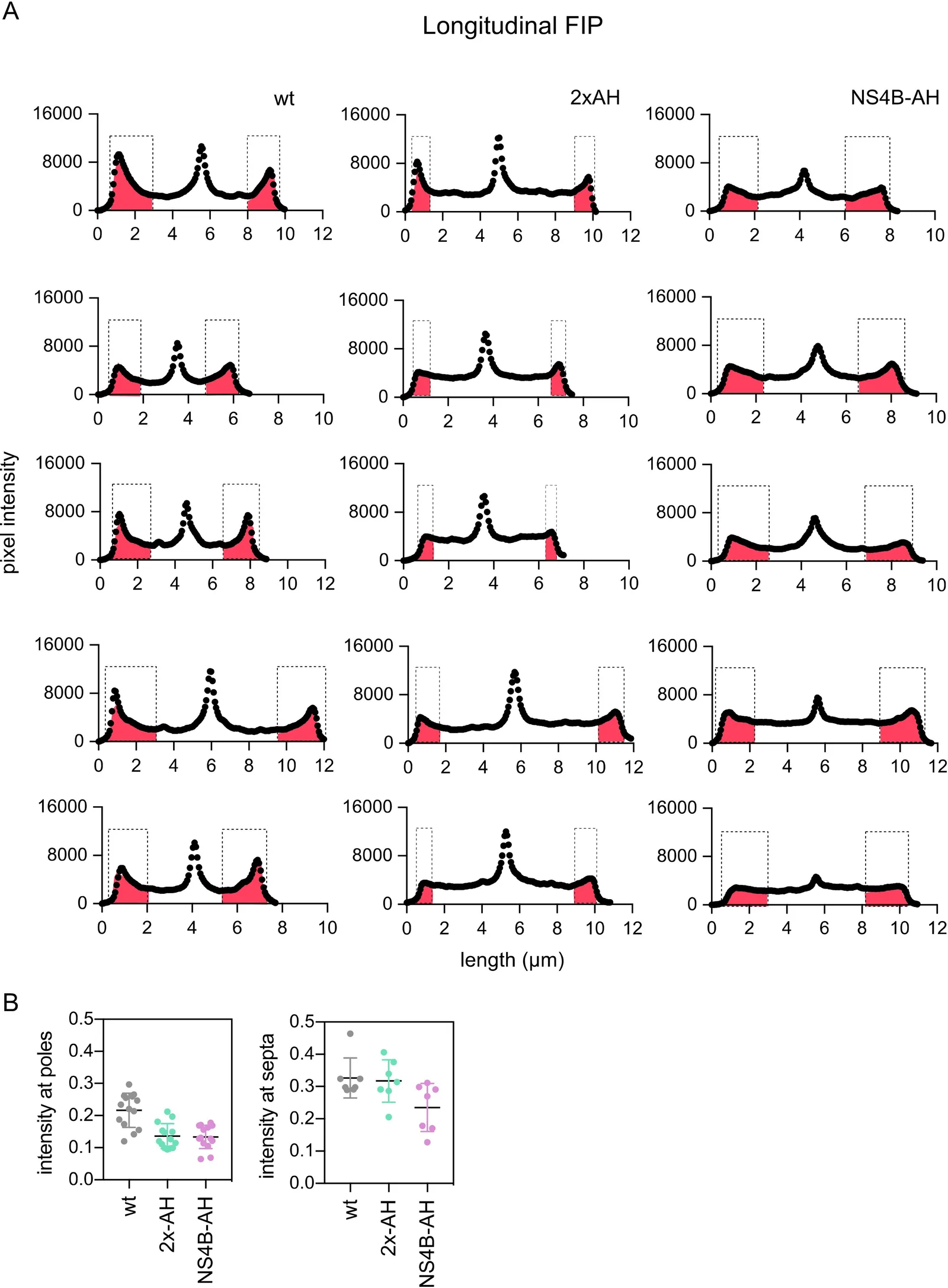
Longitudinal fluorescence intensity profiles (FIPs) of monomeric GFP (mGFP)-MinD with different membrane anchors. (**A**) Additional examples of longitudinal FIPs from cells expressing mGFP-MinD variants with different membrane affinities shown in Figure 5. Polar and septal areas were selected manually based on the fluorescence gradient. Polar areas are indicated in red. (**B**) Calculated pixel intensities and median values at poles and septa in the longitudinal FIP relative to the integrated area of intensity. Figure 5—figure supplement 1—source data 1.Fluorescence intensity data Figure 5—figure supplement 1.

To confirm that the tandem amphipathic helix increased the membrane affinity of MinD, we tested its sensitivity to membrane depolarization. Previously, it has been shown that binding of the MinD amphipathic helix to the cell membrane requires the presence of the membrane potential (Strahl and Hamoen, 2010). Dissipation of the proton motive force by the specific proton-ionophore carbonyl cyanide *m*-chlorophenylhydrazone (CCCP) did not affect the membrane localization of the tandem amphipathic helix fusion, whereas CCCP quickly disturbed the membrane attachment of wild-type MinD (Appendix 1—figure 3).

We also tested what happens with the MinD gradient when its membrane affinity is reduced. For this, the well-studied membrane binding amphipathic helix 2 from the Hepatitis C virus protein NS4B was chosen (Gouttenoire et al., 2009; Adams et al., 2015), which is hardly able to attach mGFP to the cell membrane (Figure 4). Replacing the wild-type membrane targeting helix of MinD by the weak NS4B amphipathic helix reduced the membrane association of mGFP-MinD (Figure 5D), as well as its septal and polar localization (Figure 5C, Figure 5—figure supplement 1). Interestingly, this mutant still displayed a longitudinal protein gradient. Together, these data suggest that a reversible membrane association is important for the formation of a MinD gradient along the cell axis.

### Effect of MinD membrane affinity on MinC recruitment and activity

**Figure 6.**
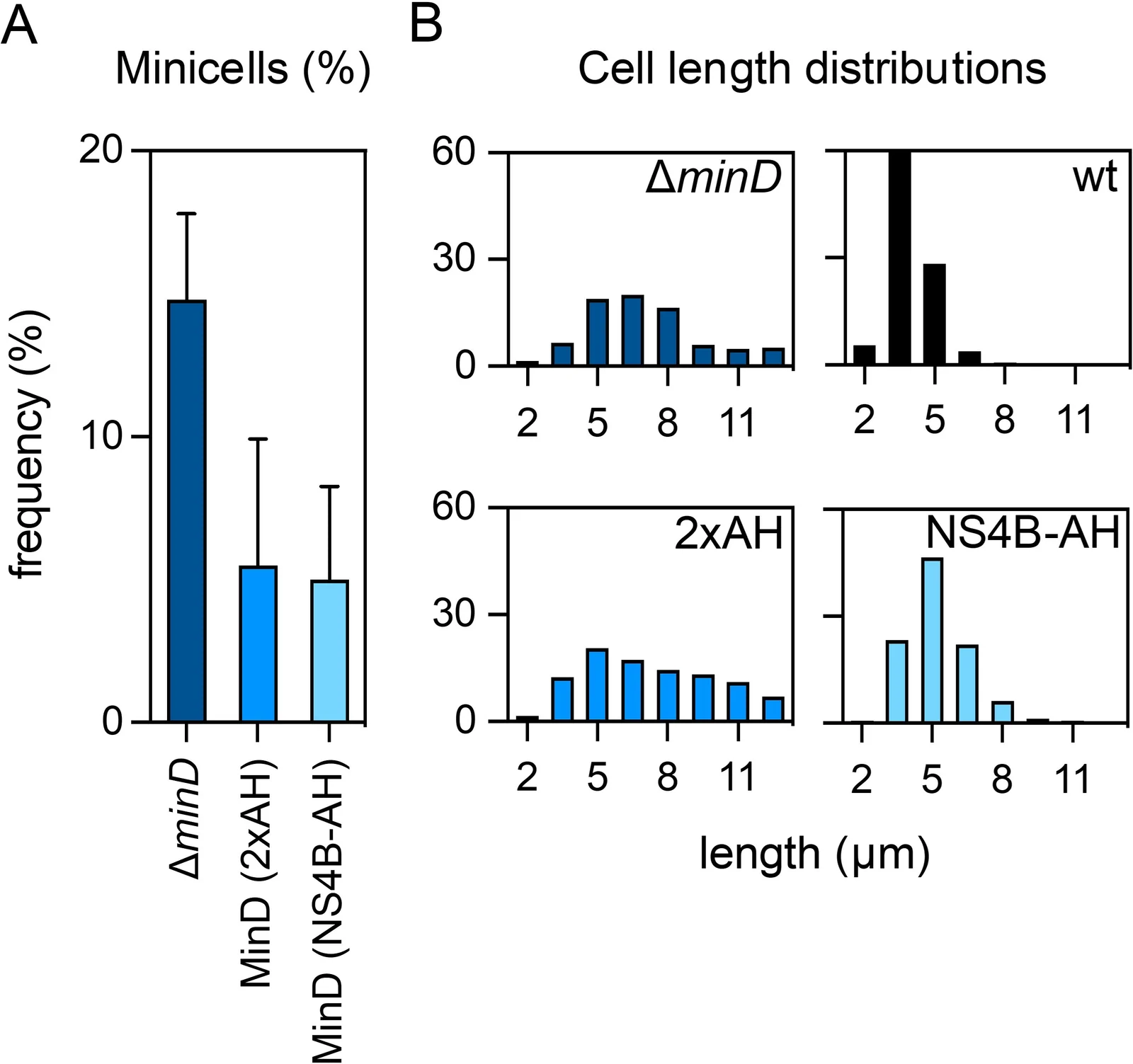
Functionality of MinD with different membrane affinities. (**A**) Minicell formation in cells expressing monomeric GFP (mGFP)-MinD with different membrane affinities. The MinD variants were expressed in a Δ*minD* background (n>300). Standard deviations are indicated. (**B**) Cell length distributions of the different strains (n>300). Strains used: 1901 (Δ*minD*), LB249 (wild-type MinD), LB507 (2xAH), and LB508 (NS4B-AH). Figure 6—source data 1.Minicell and cell length data Figure 6.

**Figure 7.**
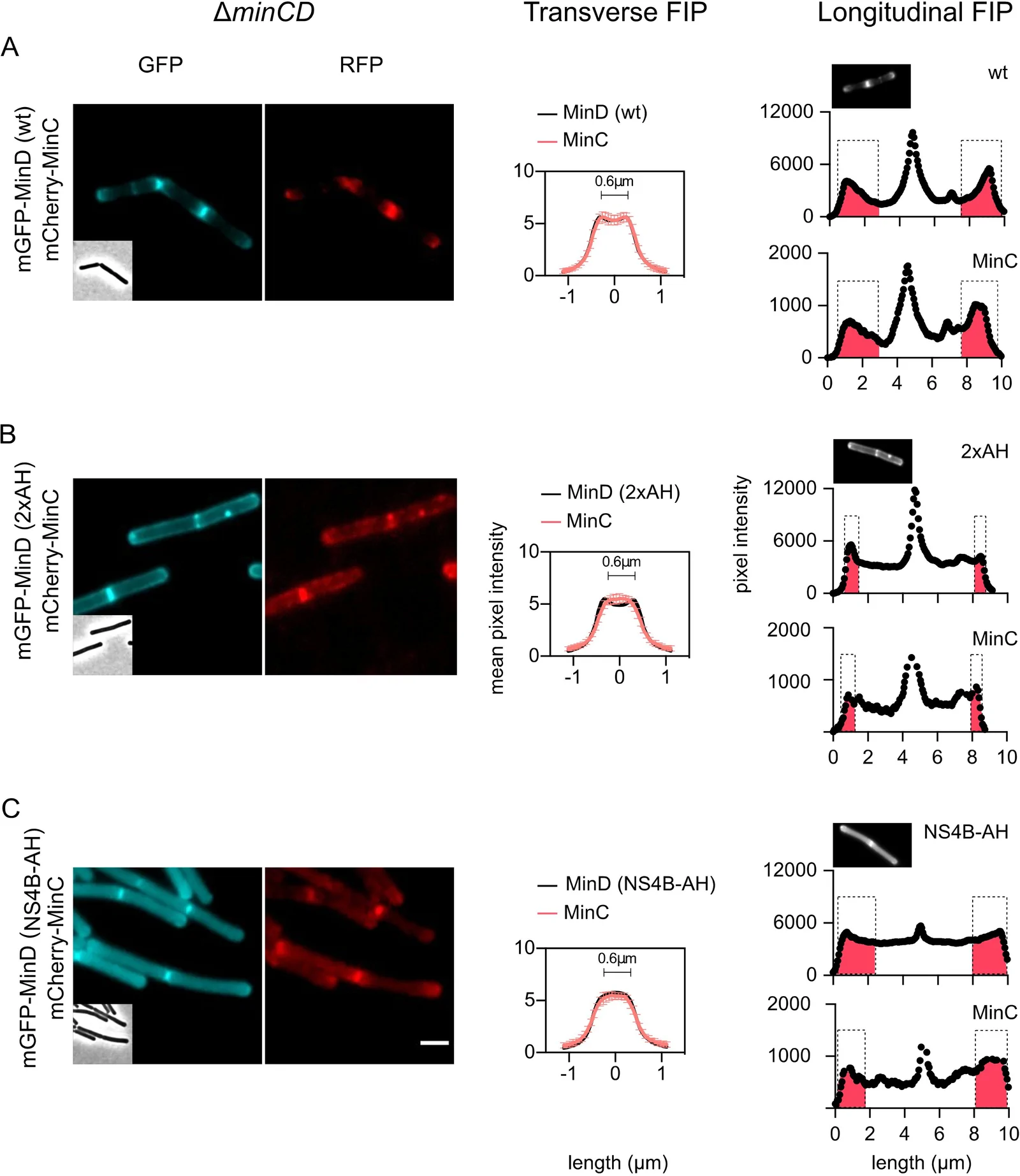
Increased MinD membrane affinity affects MinC recruitment. Fluorescence microscopy images of cells expressing monomeric GFP (mGFP)-MinD (cyan) and mCherry-MinC (red), with either the native membrane anchor (**A**), the tandem amphipathic helix (**B**), or the weak Hepatitis C virus protein NS4B-derived amphipathic helix (**C**). Corresponding phase contrast images are shown in insets. Scale bar is 2 μm. Transverse fluorescence intensity profiles (FIPs) with standard deviations are shown in the right panels (n>30). Right panels depict the longitudinal FIPs with manually shaded red areas to highlight the polar gradients. Additional examples are shown in Figure 7—figure supplement 1. The mGFP-MinD variants and mCherry-MinC were expressed in a Δ*minCD* background strain. Strains used: (**A**) LB318, (**B**) LB584, (**C**) LB559. Figure 7—source data 1.Fluorescence intensity data Figure 7.

The results shown in Figure 3 indicated that the attachment of the MinD dimer to the membrane is essential for the activity of MinC. When this attachment was strengthened by using the tandem amphipathic helix, the respective strain regularly featured additional septa, resulting in minicells (Figure 5B, red arrows), although the frequency was lower compared to a ∆*minD* mutant (Figure 6A). The strain also exhibited an elongated cell phenotype that was comparable to that of the *minD* deletion strain (Figure 6B). As shown in Figure 7B, the mGFP-MinD(2xAH) variant is capable of recruiting mCherry-MinC along the whole-cell membrane, which likely explains the elongated cell phenotype.

**Figure 7—figure supplement 1.**
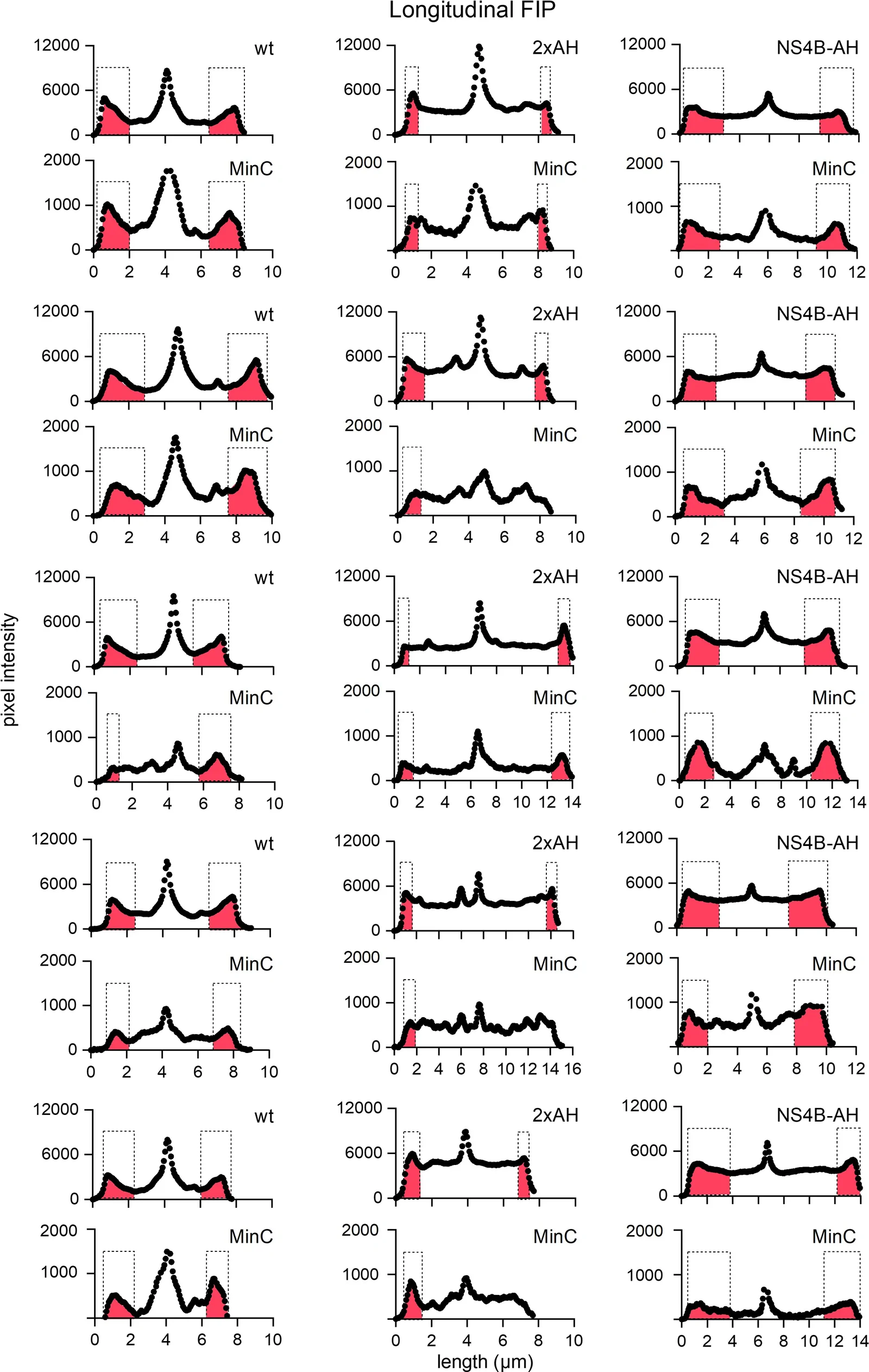
Longitudinal fluorescence intensity profiles (FIPs) of mCherry-MinC in cells expressing different monomeric GFP (mGFP)-MinD membrane-binding variants. Additional examples of longitudinal FIPs from cells expressing mCherry-MinC and mGFP-MinD variants with different membrane affinities are shown in Figure 7. Upper FIPs and lower FIPs depict MinD and MinC gradients, respectively. The latter is indicated by ‘MinC’. Figure 7—figure supplement 1—source data 1.Fluorescence intensity data Figure 7—figure supplement 1.

Weakening the membrane affinity of mGFP-MinD, by replacing the wild-type amphipathic helix with the NS4B amphipathic helix, also resulted in occasional minicell formation, but again not as numerous as in a *minD* deletion strain (Figure 6A). The cell length distribution of the strain expressing the mGFP-MinD(NS4B-AH) variant was closer to the wild-type strain (Figure 6B). When mCherry-MinC was co-expressed in this strain background, also the MinC fusion protein showed a clear fluorescent gradient along the cell axis (Figure 7C). Thus, a reversible association of MinD with the cell membrane seems to be critical to prevent misplacing the FtsZ inhibiting activity of MinC along the cell axis. Both the mGFP-MinD(2xAH) and mGFP-MinD(NS4B-AH) variants were, at least partially, able to prevent minicell formation, presumably because they are still recruited to cell poles and cell division sites through interaction with MinJ.

### Membrane gradient formation in kinetic Monte Carlo simulations

The different experiments indicated that the formation of a MinCD gradient requires the dynamic switching between monomer and dimer states of MinD (Figure 1) and a reversible MinD membrane interaction (Figure 5). So far, in silico simulations of the *B. subtilis* Min gradient have assumed a strong difference in membrane interaction between the monomeric and dimeric state of MinD, which is based on properties of the *E. coli* Min system (Howard, 2004; Feddersen et al., 2021). However, our data suggests that for *B. subtilis*, these affinities do not differ much (Figure 1). To investigate more closely whether a difference in membrane affinity between monomer and dimer is required for the formation of a MinD gradient in *B. subtilis*, we resorted to kinetic Monte Carlo simulations (see Materials and methods for more details). The Monte Carlo algorithm attempts to move one molecule at a time by a small amount in a three-dimensional space. The new interaction energy experienced by the particle is then calculated and compared with its old energy, and the move is subsequently accepted or rejected according to the standard Metropolis test (Allen and Tildesley, 1987; Binder and Heermann, 2002). This procedure is used to guide the system to the correct Boltzmann distribution in equilibrium. It has been shown that this Monte Carlo dynamics corresponds well with Brownian or molecular dynamics (Whitelam and Geissler, 2007; Sanz and Marenduzzo, 2010), and can be used to examine mechanisms underlying spatiotemporal protein gradients in cells (Lenarcic et al., 2009), provided that the moves are small enough and that the acceptance probability remains high (Whitelam and Geissler, 2007).

MinD monomers were presented as spheres (hydrodynamic radius 2.5 nm) that diffuse within and along a spherocylindrical cell geometry. The following rules were implemented: (i) Diffusion along the membrane is 10-fold slower than through the cytoplasm, and (ii) the dimer, due to its larger diameter, diffuses 2-fold slower compared to the monomer in both environments, emanating from Stokes-Einstein law and a hydrodynamic radius for the dimer, which is double that of the monomer. (iii) Based on the data from Figure 3, we started out with a 10% stronger membrane affinity for the dimer. (iv) The membrane dwell time of monomers and dimers is 0.01–0.5 s and during the simulations approximately 70% of MinD molecules reside at the membrane, approaching the valley to peak values observed experimentally (Figure 1F). (v) Dimerization and monomerization are modeled as chemical reactions between a monomer and a dimer state. Baseline rates are set such that the two states are in a 1:1 ratio, based on the longitudinal FIP in Figure 1A, and the assumption that dimers are located at the polar regions and monomers along the lateral wall. In our baseline model, dimerization at the pole, membrane, and in the cytoplasm is equally likely, and is modeled in a simple way as a reaction turning a monomeric to a dimeric MinD at constant rate. (vi) The transition from dimer to monomer, stimulated by ATP hydrolysis, occurs stochastically with a half-life of approximately 1/s, which is based on the information from *E. coli* MinD (Huang et al., 2003). (vii) MinD dimers that come in close proximity to MinJ, represented as the surface area at the polar caps, will remain attached to MinJ for some time, so that approximately 25% of MinD dimers are associated with MinJ, reminiscent of the septal localization of the D40A variant (Figure 1—figure supplement 1). We simulated 10^7^ time steps, corresponding to about 100 s real time, when the system is in steady state. Simulation details are described in the Materials and methods, and a full list of parameters and their typical values considered in simulations is presented in Supplementary file 1.

**Figure 8.**
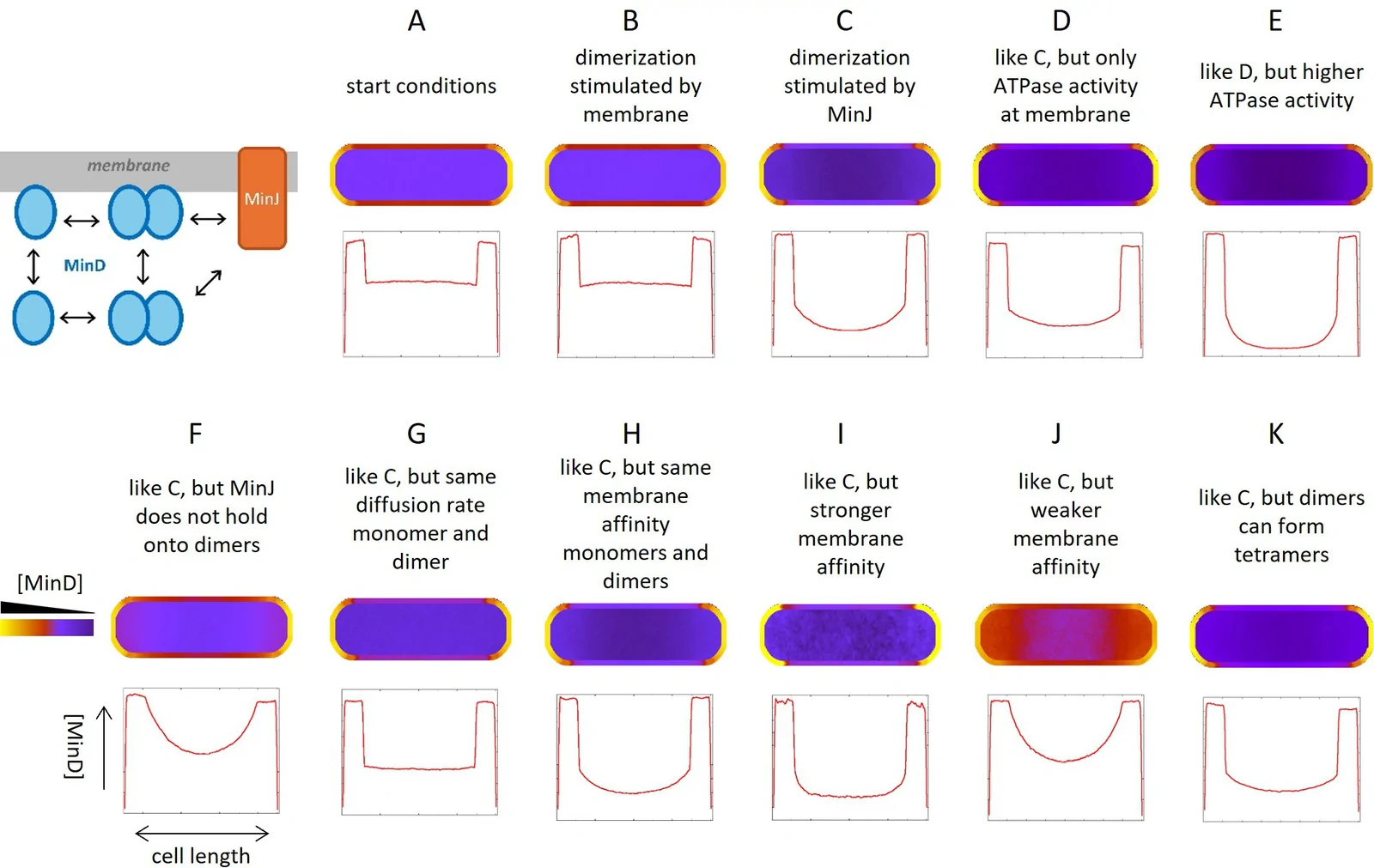
Kinetic Monte Carlo simulations of MinD localization. Whole-cell kinetic Monte Carlo simulations of MinD distribution, taking into account dimer-to-monomer transition rates (ATPase activity), membrane affinities, and MinJ interaction (schematic model). The following conditions have been simulated: (**A**) Start situation whereby (**i**) diffusion along the membrane is 10-fold slower compared to cytoplasm, (ii) MinD dimer diffuses 2-fold slower compared to the monomer in both environments, (iii) 10% stronger membrane affinity for dimer, (iv) membrane dwell time of monomers and dimers on average 1.4–4.5 s, (**v**) dimerization and monomerization rates such that dimers and monomers are approximately in a 1:1 ratio, (vi) transition from dimer to monomer occurs stochastically with a half-life of approximately 1/s, and (vii) MinD dimers in close proximity to polar regions (peak MinJ concentration) will remain attached for some time, so that approximately 25% of MinD dimers are associated with the polar caps, representing MinJ. (**B**) Same as simulation A, but membrane-attached monomers have a 2-fold higher chance of forming dimers compared to cytoplasmic monomers. (**C**) Same as simulation A, but MinJ also stimulates MinD dimerization. (**D**) Same as simulation C, but MinD ATPase activity, i.e., dimer-to-monomer transition, only occurs at the membrane. (**E**) Same as simulation D, but with a 10-fold higher ATPase activity, i.e., dimer-to-monomer transition. (**F**) Same as simulation C, but MinD dimers are not retained by MinJ. (**G**) Same as simulation C, but diffusion rates of monomer and dimer are the same. (**H**) Same as simulation C, but the membrane affinity of MinD monomers and dimers is the same. (**I**) Same as simulation C, but membrane affinity of MinD is stronger, such that the diffusion is a further 10-fold slower on the membrane. (**J**) Same as simulation C, but membrane affinity of MinD is 2-fold weaker. (**K**) Same as simulation C, but now MinD dimers can also form tetramers. Graphs indicate the average lateral projection of MinD in simulated cells.

When we applied these rules and ran the simulation, a strong accumulation of MinD at the poles was observed, but no gradient (Figure 8A). In this simulation, the chance that MinD can form a dimer is the same for monomers in the cytoplasm and those that are attached to the membrane. However, it is likely that dimer formation in the latter situation is more likely since monomers attached to the membrane by their C-terminal amphipathic helix will diffuse slower and therefore spend more time in close proximity and, importantly, they have the same spatial orientation, which will facilitate dimerization. However, when we repeated the simulation and implemented that membrane-attached monomers have a twofold higher chance of forming dimers than monomers in the cytoplasm, the localization pattern did not change (Figure 8B).

Since it seems that MinJ interacts with the dimer form of MinD (Figure 2), there is the possibility that MinJ actually stimulates MinD dimerization. Interestingly, when this property was added to the simulation, a clear MinD gradient emerged along the cell length, together with a strong polar localization signal (Figure 8C), a pattern that is in good agreement with the longitudinal FIPs observed in vivo (Figure 1A). This pattern did not change when we included the condition that only membrane-attached dimers are able to revert to monomers (Figure 8D and E), in agreement with a recent report showing that the ATPase activity of MinD depends on its interaction with lipid membranes (Feddersen and Bramkamp, 2024). In the simulation of Figure 8E, we repeated the simulation of Figure 8D, but increased the ATPase activity, i.e., dimer-to-monomer transition 10-fold. This enhances MinD gradient formation.

When we performed the simulation of Figure 8C but now with the assumption that MinJ only stimulates dimerization and does not bind MinD dimers, a MinD gradient is still formed, although the strong accumulation at the polar caps was abolished (Figure 8F). Subsequently, we repeated the simulation of Figure 8C under conditions whereby there was no difference in the diffusion rate of monomer and dimer (Figure 8G). In this case, the MinD gradient was completely lost, showing that the differential diffusion of monomers and dimers is necessary to create a steady-state gradient.

Next, we tested the role of the membrane affinity in gradient formation. First, the simulation of Figure 8C was repeated without a difference between the membrane affinity of the MinD monomer and dimer (Figure 8H). Interestingly, under these conditions, the MinD protein gradient is still being formed. When we repeated the simulation of Figure 8C and kept the 10% difference in membrane affinity between MinD monomer and dimer, but made their membrane affinities stronger, the gradient was reduced (Figure 8I), in good agreement with our in vivo observation with a MinD variant containing a tandem membrane anchor (Figure 5B). We then tested what happens when the simulation of Figure 8C was repeated with a weaker membrane affinity of MinD. As shown in Figure 8J, a clear gradient is formed, again in line with what was found when the membrane targeting amphipathic helix of MinD was replaced with the weaker amphipathic helix 2 from Hepatitis C virus protein NS4B (Figure 5C).

It has been shown that purified *E. coli* MinD can form large polymers in the presence of ATP (Suefuji et al., 2002) and, when bound to membranes, can stimulate multimerization (Heermann et al., 2020). Multimerization might further increase the diffusion difference between monomer and dimer, thus contributing to the formation of a protein gradient. Multimerization is challenging to model in our simulation setup, but, as an approximation, we tested what happens when MinD dimers can form tetramers. We ran a simulation whereby the baseline rates of dimer to tetramer and tetramer to dimer formations were equal. However, this did not lead to a dramatic change in MinD gradient (Figure 8K). In conclusion, our kinetic Monte Carlo simulations suggest that the formation of a MinD gradient requires: (i) a reversible and not too strong interaction of MinD with the membrane, (ii) a clear difference between the diffusion rates of monomer and dimer, (iii) an ATPase-driven dimer-monomer cycle, and (iv) the stimulation of dimerization at the cell poles and division sites. Importantly, a difference in membrane affinity between monomer and dimer is not required.

## Discussion

### Protein gradients in bacteria

Due to their small size, proteins diffuse rapidly throughout bacterial cells, in a matter of seconds, yet stable protein gradients can be found in a large variety of bacteria. What these systems have in common, including the MinD gradient, is a locally reduced diffusion rate of the proteins involved, which allows stable protein concentration gradients to form. The viscosity of the cell membrane is roughly 100 times larger compared to the cytoplasm (Mika et al., 2016; Mullineaux et al., 2006), and the Min system uses attachment to the cell membrane to slow down diffusion. Another approach to reduce diffusion is to bind to DNA, which occurs in the ParABS system involved in chromosome segregation in the asymmetric bacterium *Caulobacter crescentus* (Ptacin et al., 2010). This system consists of the ParA ATPase, which belongs to the same ATPase family as MinD (Leipe et al., 2002), and ParB, which binds to *parS* DNA-binding sites. The ParA dimers associate with DNA nonspecifically, whereby ParB stimulates ATP hydrolysis of ParA dimers, resulting in the dissociation from DNA. The localized dissociation and subsequent increased diffusion rate result in a ParA concentration gradient away from the *parS* locus (Surovtsev et al., 2016). The cyanobacterium *Synechococcus elongatus* uses the ParA-like protein McdA to position carboxysomes along the longitudinal axis. In this system, the protein McdB, which interacts with carboxysomes, stimulates McdA ATPase activity and its release from DNA, resulting in an oscillation of McdA over the nucleoid (MacCready et al., 2018). Recently, it was described that *Staphylococcus aureus* trigger factor (TF) forms a gradient by interacting with the cell division protein FtsK, resulting in an increased TF concentration toward the septal region (Veiga et al., 2023). How TF reduces its diffusion in *S. aureus* cells is still unclear but might be a consequence of the fact that the chaperone directly interacts with ribosomes (Deeng et al., 2016), which diffuse much slower due to their size and association with mRNA and nascent polypeptide chains.

### Previous MinD gradient modeling studies

As mentioned in the Introduction, there are many mathematical models describing the oscillating MinD gradient of *E. coli* but so far only two mathematical models focused on the MinD gradient of *B. subtilis* (Howard, 2004; Feddersen et al., 2021). Both models make use of the reversible interaction of MinD with the cell membrane. The first study, almost 20 years ago, modeled the localization of DivIVA and MinD by a reaction-diffusion system in one spatial dimension (Howard, 2004). At that time, neither MinJ nor the localization mechanism of DivIVA were known. In this early study, both MinD and DivIVA were assumed to cycle between the membrane and the cytosol, and the membrane attachment of DivIVA would depend on MinD, whereas the membrane attachment of the latter would be stabilized at the cell poles by DivIVA. However, we now know that DivIVA binds to the membrane by itself, and there is no interaction between MinD and DivIVA (Lenarcic et al., 2009).

The ATPase-stimulated monomer-dimer cycle of MinD was not included in this early model, but it was used in a recent study that employed a minimal reaction-diffusion system in three-dimensional cellular space (Feddersen et al., 2021). In this model, *B. subtilis* MinD cycles between the cell membrane and cytosol, whereby the membrane-detached MinD is in the ADP-bound state and can only rebind after exchanging ADP for ATP, like in *E. coli*. By assuming that the membrane recruitment rate is higher at the poles and septa due to the presence of MinJ or due to a kinetic increase of diffusional impacts with the membrane due to the curved geometry at poles, a steep MinD gradient could be simulated (Feddersen et al., 2021).

### New data on MinD localization

Here, we have shown that monomeric MinD, both in its ATP-bound and unbound state, has a membrane affinity that is comparable to that of the dimeric form of MinD, which is clearly different from *E. coli* MinD that requires dimerization for membrane association. In fact, it has been shown that the C-terminal membrane-targeting amphipathic helix of *E. coli* is considerably weaker than that of *B. subtilis* MinD, and that stable membrane binding is only possible when a dimer is formed so that there are two membrane-targeting domains (Szeto et al., 2003). Our findings are in good agreement with a recent report showing a strong membrane affinity for purified monomeric *B. subtilis* MinD for liposomes, independent of the presence of ATP (Feddersen and Bramkamp, 2024).

We have also shown that MinJ is not required for the membrane recruitment of MinD (Figure 2). We have no explanation for the differences between our findings and that of the previous studies in which it has been shown that MinJ is needed for the membrane localization of MinD (Bramkamp et al., 2008; Feddersen et al., 2021). However, since the membrane-targeting amphipathic alpha helix of *B. subtilis* MinD is by itself sufficient to recruit GFP to the cell membrane, even in *E. coli*, which does not contain a MinJ homologue (Szeto et al., 2003; Strahl and Hamoen, 2010), it is difficult to envision how the absence of MinJ would bring MinD to the cytosol.

In another study, it has been suggested that the C-terminal amphipathic helix of MinD by itself is sufficient to recruit GFP to cell division sites, even in the absence of either MinJ or DivIVA (Ishikawa et al., 2017). However, as indicated in Figure 1, the presence of two perpendicular-observed membranes that are formed during septum synthesis gives a higher fluorescence membrane signal, which might have been mistakenly interpreted as septal localization. In addition, in our hands, a GFP fusion with the C-terminal amphipathic helix of *B. subtilis* MinD shows a clear fluorescence membrane stain but no accumulation at division sites (Figure 4B).

### Kinetic Monte Carlo model

Our in vivo findings warranted a new mathematical model to study the formation of the *B. subtilis* MinD gradient (see Materials and methods for more details). The kinetic Monte Carlo model showed that a membrane attachment-detachment cycle is not required to form a protein gradient, but that a dynamic ATP-driven cycle between monomer and dimer is crucial, coupled to the differences in diffusion rates. We have shown that the MinD dimer associates with MinJ, but this by itself, even when the interaction was reversible, was not enough to create a gradient. We ascribe this to the fact that both monomers and dimers bind to the membrane with small differences in affinities in our model, whereas in Feddersen et al., 2021, the monomer state is not membrane-associated. To establish a gradient in our simulations, we needed to include the requirement that MinJ stimulates and/or stabilizes MinD dimerization, but this MinJ activity remains speculative. It should be stressed that MinJ is not crucial for MinD dimerization, since a *minJ* mutant shows a strong filamentous phenotype that can be suppressed by a *minC* deletion, indicating that MinD dimerization, required for MinC activity, also occurs in the absence of MinJ.

Although a difference in membrane affinity between monomer and dimer is not necessary to establish a gradient, the modeling shows that a transient and reversible membrane binding of MinD is necessary. This was also confirmed by the reduced formation of a gradient when the membrane affinity of MinD was increased by using a tandem C-terminal amphipathic helix (Figure 5B). This can be explained by considering the emergent length scale over which the gradient develops. As in reaction-diffusion models for morphogenesis, this length scale is given by the square root of a diffusion coefficient over a reaction rate. Our simulations suggest that a possible formula is given by $l \sim \sqrt{\frac{D_{m}-D_{d}}{k_{m}}}$ , with $D_{d}$ and $D_{m}$ the dimer and monomer diffusion (the relevant rate is that on the membrane), and $k_{m}$ the rate of conversion between dimers and monomers. Increasing the interaction with the membrane leads to a decrease in diffusivity, and concomitantly a reduction in the length scale over which the gradient can be observed.

In our modeling, MinJ is simply represented by the polar cap area. FRAP studies have shown that the recruitment of MinJ to cell poles and division sites is quite dynamic, with a fluorescence recovery rate close to a minute (Feddersen et al., 2021). However, the fluorescence recovery rate of MinD is approximately 8-fold higher (Feddersen et al., 2021). Because of this large difference in diffusion rates, we have ignored the diffusion of MinJ in our modeling. We do not foresee different results if we were to include MinJ diffusion in our Monte Carlo simulations, provided that in steady state MinJ is on average localized at the poles.

Another aspect that we have only partially addressed in the simulations is MinD polymerization. It has been shown that purified *E. coli* MinD can form large polymers in the presence of ATP (Suefuji et al., 2002), and, when bound to membranes, can stimulate multimerization (Heermann et al., 2020). In our simulations, we tested the effect of MinD tetramerization; however, this did not improve MinD gradient formation (Figure 8K). It should be mentioned that formation of large polymers was not found with purified *B. subtilis* MinD (Wang et al., 2022). Moreover, FRAP experiments with *B. subtilis* show a fluorescence recovery rate of seconds for MinD at division sites (Feddersen et al., 2021), suggesting that if such polymers exist, they are not very stable. Thus, it remains to be seen whether MinD multimerization is relevant in *B. subtilis* cells.

In conclusion, we have shown here that a dynamic cyclic change in membrane affinity of MinD is not required for the formation of a concentration gradient in the cell. Instead, our modeling suggests that stimulation of MinD dimerization at cell poles and cell division sites is needed. It will be interesting to see whether future experiments prove this suggestion right or wrong.

## Materials and methods

**Key resources table:** 

Reagent type (species) or resource	Designation	Source or reference	Identifiers	Additional information
Strain, strain background (*B. subtilis*)	168	Barbe et al., 2009		Wild-type
Strain, strain background (*B. subtilis*)	1901	Marston et al., 1998		*minD::erm*
Strain, strain background (*B. subtilis*)	3381	van Baarle and Bramkamp, 2010		*minC::km*
Strain, strain background (*B. subtilis*)	3309	van Baarle and Bramkamp, 2010		*minCD::km*
Strain, strain background (*B. subtilis*)	RD021	Bramkamp et al., 2008		*minJ::tet*
Strain, strain background (*B. subtilis*)	LB249	This paper		*minD::erm amyE::spc Pxyl-mGFP-minD*
Strain, strain background (*B. subtilis*)	LB250	This paper		*minD::erm amyE::spc Pxyl-mGFP-minD(K16A*)
Strain, strain background (*B. subtilis*)	LB251	This paper		*minD::erm amyE::spc Pxyl-mGFP-minD(G12V*)
Strain, strain background (*B. subtilis*)	LB252	This paper		*minD::erm amyE::spc Pxyl-mGFP-minD(D40A*)
Strain, strain background (*B. subtilis*)	LB305	This paper		*minCD::km amyE::spc Pxyl-mGFP-minD*
Strain, strain background (*B. subtilis*)	LB306	This paper		*minCD::km amyE::spc Pxyl-mGFP-minD(K16A*)
Strain, strain background (*B. subtilis*)	LB307	This paper		*minCD::km amyE::spc Pxyl-mGFP-minD(G12V*)
Strain, strain background (*B. subtilis*)	LB308	This paper		*minCD::km amyE::spc Pxyl-mGFP-minD(D40A*)
Strain, strain background (*B. subtilis*)	LB318	This paper		*minCD::km amyE::spc Pxyl-mGFP-minD aprE::cat Pspac-mCherry-minC*
Strain, strain background (*B. subtilis*)	LB319	This paper		*minCD::km amyE::spc Pxyl-mGFP-minD(K16A) aprE::cat Pspac-mCherry-minC*
Strain, strain background (*B. subtilis*)	LB320	This paper		*minCD::km amyE::spc Pxyl-mGFP-minD(G12V) aprE::cat Pspac-mCherry-minC*
Strain, strain background (*B. subtilis*)	LB321	This paper		*minCD::km amyE::spc Pxyl-mGFP-minD(D40A) aprE::cat Pspac-mCherry-minC*
Strain, strain background (*B. subtilis*)	LB405	This paper		*minCD::km amyE::spc Pxyl-mGFP-minD*
Strain, strain background (*B. subtilis*)	LB406	This paper		*minCD::km amyE::spc Pxyl-mGFP-minD(K16A*)
Strain, strain background (*B. subtilis*)	LB407	This paper		*minCD::km amyE::spc Pxyl-mGFP-minD(G12V*)
Strain, strain background (*B. subtilis*)	LB408	This paper		*minCD::km amyE::spc Pxyl-mGFP-minD(D40A*)
Strain, strain background (*B. subtilis*)	LB409	This paper		*minCD::km minJ::tet amyE::spc Pxyl-mGFP-minD*
Strain, strain background (*B. subtilis*)	LB410	This paper		*minCD::km minJ::tet amyE::spc Pxyl-mGFP-minD(K16A*)
Strain, strain background (*B. subtilis*)	LB411	This paper		*minCD::km minJ::tet amyE::spc Pxyl-mGFP-minD(G12V*)
Strain, strain background (*B. subtilis*)	LB412	This paper		*minCD::km minJ::tet amyE::spc Pxyl-mGFP-minD(D40A*)
Strain, strain background (*B. subtilis*)	LB507	This paper		*minD::erm amyE::spc Pxyl-mGFP-minD-BsMTS-tandem(2xAH with linker in between*)
Strain, strain background (*B. subtilis*)	LB508	This paper		*minD::erm amyE::spc Pxyl-mGFP-minD-HCV-NS4B-AH*
Strain, strain background (*B. subtilis*)	LB559	This paper		*amyE::spc Pxyl-mGFP-minD-HCV-NS4B-AH aprE::cat Pspac-mCherry-minC minCD::km*
Strain, strain background (*B. subtilis*)	LB584	This paper		*amyE::spc Pxyl-mGFP-minD-BsMTS-tandem(2xAH with linker in between) aprE::cat Pspac-mCherry-minC minCD::km*
Strain, strain background (*B. subtilis*)	FBB043	This paper		*amyE::spc Pxyl-GFP-AH(MinD*)
Strain, strain background (*B. subtilis*)	FBB053	This paper		*amyE::spc Pxyl-GFP-BsMTS-tandem(MinD*)
Strain, strain background (*B. subtilis*)	FBB046	This paper		*amyE::spc Pxyl-GFP-AH(NS4B*)
Strain, strain background (*B. subtilis*)	LB609	This paper		*amyE::spc Phyperspank-sfGFP (from pDR111-N015-sfGFP*)
Strain, strain background (*B. subtilis*)	LB643	This paper		*amyE::spc Pxyl-mGFP-minD(I260E) aprE::cat Pspac-mCherry-minC minCD::km*
Strain, strain background (*B. subtilis*)	LB644	This paper		*amyE::spc Pxyl-mGFP-minD(D40A I260E) aprE::cat Pspac-mCherry-minC minCD::km*
Recombinant DNA reagent	pAPNC213	Morimoto et al., 2002	Plasmid	*bla aprE3’ spc Pspac-lacI aprE5’*
Recombinant DNA reagent	pDR111-N015-sfGFP	Nordholt et al., 2017	Plasmid	*bla amyE3’ spc Phyperspank-lacI-sfGFP amyE5’*
Recombinant DNA reagent	pHJS113	Strahl and Hamoen, 2010	Plasmid	*bla amyE3’ spc Pxyl-GFP-minD(K16A) amyE5’*
Recombinant DNA reagent	pSG1729	Lewis and Marston, 1999	Plasmid	*bla amyE3’ spc Pxyl-GFP amyE5’*
Recombinant DNA reagent	pSG1730	Marston et al., 1998	Plasmid	*bla amyE3’ spc Pxyl-GFP-minD amyE5’*
Recombinant DNA reagent	pSG2	Fort and Errington, 1985	Plasmid	*bla cat*
Recombinant DNA reagent	pSS153	Syvertsson et al., 2021	Plasmid	*bla aprE3’ Phag-mCherry-cat aprE5’*
Recombinant DNA reagent	pAPNCcat	This paper	Plasmid	*bla aprE3’ cat Pspac-lacI aprE5’*
Recombinant DNA reagent	pHJS112	This paper	Plasmid	*bla aprE3’ cat Pspac-mCherry aprE5’*
Recombinant DNA reagent	pHJS115	This paper	Plasmid	*bla amyE3’ spc Pxyl-GFP-minD(G12V) amyE5’*
Recombinant DNA reagent	pHJS116	This paper	Plasmid	*bla amyE3’ spc Pxyl-GFP-minD(D40A) amyE5’*
Recombinant DNA reagent	pHJS117	This paper	Plasmid	*bla amyE3’ spc Pxyl-GFP-MTS(minD) amyE5’*
Recombinant DNA reagent	pHJS119	This paper	Plasmid	*bla amyE3’ spc Pxyl-GFP-MTS(minD)-tandem (with linker in between) amyE5’*
Recombinant DNA reagent	pHJS121	This paper	Plasmid	*bla amyE3’ spc Pxyl-GFP-NS4B-AH amyE5’*
Recombinant DNA reagent	pHJS123	This paper	Plasmid	*bla amyE3’ spc Pxyl-GFP-minD-BsMTS-tandem (with linker in between) amyE5’*
Recombinant DNA reagent	pHJS125	This paper	Plasmid	*bla amyE3’ spc Pxyl-GFP-minD-NS4B-AH amyE5’*
Recombinant DNA reagent	pLB11	This paper	Plasmid	*bla aprE3’ cat Pspac-mCherry-minC aprE5’*
Recombinant DNA reagent	pLB21	This paper	Plasmid	*bla amyE3’ spc Pxyl-mGFP-minD amyE5’*
Recombinant DNA reagent	pLB22	This paper	Plasmid	*bla amyE3’ spc Pxyl-mGFP-minD(K16A) amyE5’*
Recombinant DNA reagent	pLB23	This paper	Plasmid	*bla amyE3’ spc Pxyl-mGFP-minD(G12V) amyE5’*
Recombinant DNA reagent	pLB24	This paper	Plasmid	*bla amyE3’ spc Pxyl-mGFP-minD(D40A) amyE5’*
Recombinant DNA reagent	pLB49	This paper	Plasmid	*bla amyE3’ spc Pxyl-mGFP-minD-BsMTS-tandem(with linker in between) amyE5’*
Recombinant DNA reagent	pLB50	This paper	Plasmid	*bla amyE3’ spc Pxyl-mGFP-minD-NS4B-AH amyE5’*
Recombinant DNA reagent	pLB71	This paper	Plasmid	*bla amyE3’ spc Pxyl-mGFP-minD(I260E) amyE5’*
Recombinant DNA reagent	pLB72	This paper	Plasmid	*bla amyE3’ spc Pxyl-mGFP(D40A I260E)-minD amyE5’*
Sequence-based reagent	LB1	This paper	PCR primers	GCGCGGGATCCATGAAGACCAAAAAGCAGCAATATG
Sequence-based reagent	LB2	This paper	PCR primers	CGCGCGAATTCTCACATTCCTCCCTCAAGCCTTG
Sequence-based reagent	HS05	This paper	PCR primers	GCTAATTTTATTGCAATAACAGGTG (To linearize pAPNC213)
Sequence-based reagent	HS06	This paper	PCR primers	GACCGTTAGCGTTTAAGTACATC (To linearize pAPNC213)
Sequence-based reagent	HS07	This paper	PCR primers	TAAACGCTAACGGTCCAGTAATATTGACTTTTAAAAAAGG (*cat* cassette)
Sequence-based reagent	HS08	This paper	PCR primers	TGCAATAAAATTAGCTTATAAAAGCCAGTCATTAGGCC (*cat* cassette)
Sequence-based reagent	HS437	This paper	PCR primers	GCGCGGTCGACACATAAGGAGGAACTACTATGGTC (mCherry)
Sequence-based reagent	HS438	This paper	PCR primers	(mCherry)
Sequence-based reagent	HS508	This paper	PCR primers	CACAGAATAGTCTTTTAAGTAAGTC (*aprE*)
Sequence-based reagent	HS509	This paper	PCR primers	CCGGAACATCAGGATGCTGAC (*aprE*)
Sequence-based reagent	HS410	This paper	PCR primers	CCTGTCCACACAATCTAAACTTTCGAAAGATCCC (A206K mutation in GFP)
Sequence-based reagent	HS411	This paper	PCR primers	GGGATCTTTCGAAAGTTTAGATTGTGTGGACAGG (A206K mutation in GFP)
Sequence-based reagent	HS112	This paper	PCR primers	GCGGAGTAGGTGCGACAACAACATCTG (K16A mutation in MinD)
Sequence-based reagent	HS113	This paper	PCR primers	CAGATGTTGTTGTCGCACCTACTCCGC (K16A mutation in MinD)
Sequence-based reagent	HS205	This paper	PCR primers	GGAATGATGGCTAAGGAAAAGTCATTTTTCGG (I260E mutation in MinD)
Sequence-based reagent	HS206	This paper	PCR primers	CCGAAAAATGACTTTTCCTTAGCCATCATTCC (I260E mutation in MinD)
Sequence-based reagent	HS320	This paper	PCR primers	CTTCGGGAAAAGTGGGAGTAGGTAAG (G12V mutation in MinD)
Sequence-based reagent	HS321	This paper	PCR primers	CTTACCTACTCCCACTTTTCCCGAAG (G12V mutation in MinD)
Sequence-based reagent	HS322	This paper	PCR primers	GCTTAGTAGATACTGCGATAGGACTGCGC (D40A mutation in MinD)
Sequence-based reagent	HS323	This paper	PCR primers	GCGCAGTCCTATCGCAGTATCTACTAAGC (D40A mutation in MinD)
Sequence-based reagent	FB135	This paper	PCR primers	CTTCGGATCCACGGGCCCCCCCTCGAGTTGGGTGAGGCTATCGTAATAAC (*minD*)
Sequence-based reagent	FB136	This paper	PCR primers	CTAGAACTAGAATTCTTAAGATCTTACTCCGAAAAATG (MinD MTS)
Sequence-based reagent	FB149	This paper	PCR primers	CTTCGGATCCACGGATCAGGAGTGCTTGAAGAGCAAAACAAAGG (MinD MTS)
Sequence-based reagent	FB138	This paper	PCR primers	CTAGAACTAGAATTCTTATGATCTCACTCCAAAAAATGATTTAATTTTCGCCATCATTCCTTTGTTTTGTTCTTCCAGCACTCCTCCAGATCTTACTCCGAAAAATGAC (MinD MTS with 2xAH with linker)
Sequence-based reagent	FB141	This paper	PCR primers	CTAGAACTAGAATTCTTAAATCCATTGGTGCAGTCTTCTCAGCAGTTGTGTCACTGTCAGTGATGACAGAATGCCGTTTTGCTCTTCAAGCACCTG (HCV-NS4B-AH)

### Bacterial strains, growth conditions, and media

Luria-Bertani (LB) agar was used for the routine selection and maintenance of both *B. subtilis* and *E. coli* strains. For *B. subtilis*, cells were grown in LB medium or Spizizen minimal salt medium supplemented with 0.05% yeast extract (SMMY). SMMY consists of 2 g/l (NH_4_)_2_SO_4_, 14 g/l K_2_HPO_4_, 6 g/l KH_2_PO_4_, 1 g/l sodium citrate, 2 g/l MgSO_4_, 5 g/l fructose, 2 g/l tryptophan, 0.2 g/l casamino acids, and 2.2 g/l ammonium ferric citrate. All strains were grown at 37°C. The supplements were added as required: 100 µg/ml ampicillin, 5 µg/ml chloramphenicol, 5 µg/ml kanamycin, 100 µg/ml spectinomycin, 10 µg/ml tetracycline, and 1 µg/ml erythromycin. The *B. subtilis* strains used are listed in the Key resources table. The mutant strains kindly provided by other labs were transformed into our laboratory strain to ensure that all strains were isogenic.

### Strain constructions

Molecular cloning, PCR, and transformations were carried out by standard techniques. Plasmids and oligonucleotides used in this study are listed in the Key resources table. The *minC*, *minD*, *minCD,* and *minJ* gene deletions were introduced from existing strains listed in the Key resources table by standard natural transformation (Hamoen et al., 2002). The *aprE*-integration vector containing mCherry under an IPTG-inducible promoter (P*spac*) was constructed as follows. The spectinomycin marker of pAPNC213 (Morimoto et al., 2002) was replaced with a chloramphenicol resistance cassette (*cat*) using In-Fusion cloning (Clonetech). For this aim, pAPNC123 was PCR-linearized with oligonucleotide pair HS05/HS06, and the *cat* cassette was amplified from pSG2 (Fort and Errington, 1985) with oligonucleotide pair HS07/HS08. These PCR products were fused together with In-Fusion cloning, resulting in pAPNC*cat*; this plasmid was verified by sequencing. Next, the mCherry encoding gene was amplified using oligonucleotide pair HS437/HS438 and plasmid template pSS153 (Syvertsson et al., 2021) as template. SalI and BamHI restriction sites were inserted into the primers. The mCherry PCR product and the pAPNC*cat* were digested with SalI and BamHI and ligated. The resulting plasmid was verified by sequencing and named pHJS112.

The IPTG-inducible mCherry-*minC* fusion was constructed as follows. A PCR fragment containing *minC* was amplified with oligonucleotide pair LB1/LB2 and genomic DNA of the wild-type strain 168 as template. BamHI and EcoRI restriction sites were inserted into the primers. The PCR product and the *aprE*-integration vector pHJS112 were digested with BamHI and EcoRI restriction enzymes and ligated. The resulting plasmid pLB11 was verified by sequencing and transformed into *B. subtilis*-competent cells, resulting in strain LB31. The *aprE* integration was verified by PCR, amplifying the genomic DNA with oligonucleotide pair HS508/HS509 and sequencing.

The *amyE*-integration vector pSG1730 containing GFP fused to *minD* under a xylose-inducible promoter (Marston et al., 1998) was used to exchange the *minD* residue glycine 12 to valine (G12V) and aspartic acid 40 to alanine (D40A) by incorporation of the corresponding mutation using the QuickChange site-directed mutagenesis reaction (Stratagene) and the oligonucleotide pairs HS320/HS321 and HS322/HS323, respectively. The resulting plasmids were verified by sequencing and named pHJS115 and pHJS116, respectively. The plasmid pHJS113 containing GFP fused to *B. subtilis minD* coding sequence with the lysine 16 to alanine (K16A) exchange under a xylose-inducible promoter was reported previously (Strahl and Hamoen, 2010).

Plasmid pSG1729 (Lewis and Marston, 1999), containing GFP under a xylose-inducible promoter, was used to generate a construct with GFP fused to the native *minD* membrane targeting amphipathic helix (KGMMAKIKSFFG). This domain was amplified from *B. subtilis* wild-type strain 168 with oligonucleotide pair FB136/FB149. EcoRI and BamHI restriction sites were inserted into the primers. The PCR product and the plasmid pSG1729 were digested with EcoRI and BamHI restriction enzymes and ligated. The new plasmid was verified by sequencing and named pHJS117. Plasmid pHJS117 was used to generate a construct with GFP fused to two *minD* amphipathic helices in tandem with a short linker of glycines in between (2xAH). This tandem membrane targeting sequence was created by amplification with oligonucleotides FB138/FB149 and pHJS117 as template. The primer FB138 carries a long overhang that generates an extra MinD membrane targeting amphipathic helix fused to the existing membrane targeting domain (KGMMAKIKSFFGVRS**GG**VLEEQNKGMMAKIKSFFG). EcoRI and BamHI restriction sites were inserted into the primers. The PCR product and the plasmid pSG1729 were digested with *Eco*RI and *Bam*HI restriction enzymes and ligated. The new plasmid was verified by sequencing and named pHJS119.

Another construct with GFP fused to the NS4B amphipathic helix 2 from Hepatitis C virus (NS4B-AH) was generated as follows. Plasmid pSG1729 was amplified with oligonucleotides FB141/FB149. The primer FB141 carries a long overhang that generates the NS4B-AH anchor (TVTQLLRRLHQWI). EcoRI and BamHI restriction sites were inserted into the primers. The PCR product and the plasmid pSG1729 were digested with EcoRI and BamHI restriction enzymes and ligated. The new plasmid was verified by sequencing and named pHJS121.

We constructed two different *amyE*-integration plasmids with GFP fused to *minD* with different membrane-targeting sequences. The native *minD* amphipathic helix was either replaced with two *minD* amphipathic helices in tandem with a short linker of glycines in between (MinD2xAH) or with the Hepatitis C virus NS4B amphipathic helix 2 (MinDNS4B-AH). The *minD* gene was amplified from the *B. subtilis* genome (strain 168) with oligonucleotide pairs FB135/FB138 and FB135/FB141, respectively. BamHI and EcoRI restriction sites were inserted into the primers. The primers FB138 and FB141 carry a long overhang that generates the 2xAH and the NS4B-AH, respectively. The digested PCR fragments were ligated with BamHI/EcoRI-linearized pSG1729, containing GFP under a xylose-inducible promoter. The new plasmids were verified by sequencing and named pHJS123 and pHJS125, respectively.

Plasmids pSG1730, pHJS113, pHJS115, pHJS116, pHJS123, and pHJS125 were used as template for the QuickChange site-directed mutagenesis reaction (Stratagene) with oligonucleotide pair HS410/HS411 to introduce the A206K exchange in the GFP coding sequence to reduce protein dimerization (mGFP). The resulting plasmids were verified by sequencing and named pLB21, pLB22, pLB23, pLB24, pLB49, and pLB50, respectively. Plasmids pLB21 and pLB24 were used to further exchange the *minD* isoleucine 260 to glutamic acid (I260E) by QuickChange site-directed mutagenesis reaction (Stratagene) with oligonucleotide pair HS205/HS206. The resulting plasmids were verified by sequencing and named pLB71 and pLB72, respectively. These constructs were integrated at the *amyE* locus in different genetic backgrounds.

### Western blot analysis

To detect the cellular levels of the different GFP-MinD variants, the strains were induced with 0.1% xylose for three doublings. 2 ml of cultures, with comparable ODs, were centrifuged, and the cell pellets were flash-frozen in liquid nitrogen. The pellets were then resuspended in 200 µl of buffer containing 10 mM Tris-HCl, pH 8, 100 mM NaCl, and 1% Triton X-100. The cells were lysed with 100 µg/ml lysozyme during 15 min incubation at room temperature. Cell debris was spun down (20 min Eppendorf centrifuge, 14,000 rpm, 4°C), and 10 μl of samples were loaded onto a 10% SDS-PAGE, followed by separation by electrophoresis at 150 V for 60 min. The proteins were then transferred to a PVDF membrane (Thermo Scientific) with transfer buffer (14.4 g/l glycine, 3 g/l Tris base, and 15% methanol) using a mini-wet electroblotting system (Bio-Rad) at 55 V for 130 min. The transferred membrane was then blocked in blocking buffer (1% skimmed milk powder in TBST buffer 50 mM Tris, 150 mM NaCl, and 0.05% Tween-20) overnight and rinsed twice for 5 min in TBST. The proteins were probed with a 1:2000 dilution of rabbit anti-GFP primary antibody (Invitrogen) in 10 ml TBST, incubated for 1 hr. The membrane was washed 3× in TBST and incubated in a 1:20,000 dilution of secondary antibody, goat anti-rabbit IR dye (LI-COR Biosciences), in 10 ml TBST during 1 hr. The immunoblot was developed using an Odyssey imaging system (LI-COR Biosciences).

### Microscopy

Microscopy was performed on an inverted fluorescence Nikon Eclipse Ti microscope equipped with a CFI Plan Apochromat DM 100× oil-immersion objective, an Intensilight HG 130 W lamp, and a C11440-22CU Hamamatsu ORCA camera. Images were analyzed using ImageJ v.1.48d5 (National Institutes of Health) software and the plugin ObjectJ (Vischer et al., 2015). To measure the transverse FIPs, line-scans perpendicular to the cell axis were marked. The polar gradient areas, marked in red in figures, were manually assigned by means of visual inspection of the fluorescence gradient of the FIPs. The membrane affinity was estimated as the ratio valley/peaks of the transverse FIP, using an average of at least 30 cells per dataset. When appropriate, data was analyzed with t-tests (p-values) using GraphPad Prism for Mac, GraphPad Software.

For microscopy, cells were grown to exponential phase by diluting 1:100 a starter culture grown overnight in liquid medium (SMMY) into fresh medium, and allowing at least three doublings before mounting on microscopy slides covered with a thin film of 1% agarose. 0.1% xylose, and 0.01 mM IPTG were used for the induction since these concentrations complemented a Δ*minCD* deletion strain, preventing minicell formation. Membranes were stained with the fluorescent membrane dye FM-95 (0.5 µg/ml final concentration), incubated for 5 min prior to mounting onto microscope slides covered with a thin film of 1% agarose. Dissipation of the proton motive force (pmf) was carried out by the addition of 100 μM CCCP for 10 min. CCCP was dissolved in DMSO (0.1% final concentration of DMSO). As control, cells were incubated with 0.1% DMSO.

Microscopy experiments were repeated several times on different days, providing sufficient biological replicates.

### Kinetic Monte Carlo simulations

To model the formation of a steady-state gradient in *B. subtilis* wild-type cells and mutants, kinetic Monte Carlo simulations were performed. Kinetic Monte Carlo is a stochastic algorithm known to be equivalent to molecular dynamics for sufficiently small local moves (Whitelam and Geissler, 2007; Sanz and Marenduzzo, 2010). The system simulated consisted of a collection of 1000 MinD proteins enclosed inside a spherocylinder, with cap diameter $2R_{cyl}$ and total height $h_{cyl}$ (we chose an aspect ratio $\frac{h_{cyl}}{2R_{cyl}}=4$, sizes 80 by 20 in simulation units) modeling a *B. subtilis* cell. MinD proteins can be in one of two states, either monomeric (M) or dimeric (D). The M and D states have both affinities for the membrane (the surface of the spherocylinder). To model this, we introduce an attractive potential, a square well with range 1 and strength $\epsilon _{M}$ and $\epsilon _{D}$ for the M and D states, respectively. Given our experimental results that the dimer is only weakly stickier than the monomer, we ran our simulations with $\epsilon _{D}=1.1\, \epsilon _{M}$ and $\epsilon _{M}$=1 k_B_T. Additionally, the D state, but not the M state, may interact with the caps (poles) in the spherocylinders, with an extra interaction again represented by a square well, with range 1 and strength $\epsilon _{C}$. This interaction simulates binding between dimeric MinD and MinJ. Various cases were considered, but results are reported, for simplicity, for two cases only: $\epsilon _{C}$=0 (no additional polar interaction) or $\epsilon _{C}$=0.7 $\epsilon _{M}$. A third parameter which varies between M and D is the diffusion coefficient: for simplicity, unless otherwise stated, we assume this to scale inversely with molecular weight, so that the diffusivity of the dimer, $D_{d}$, is half that of the monomer, $D_{m}$.

Key to the formation of the gradient is the fact that the reaction between the M and D states of MinD is not in thermodynamic equilibrium, as it is linked to ATP hydrolysis. In our model, D turns into M, due to ATP hydrolysis, at a constant rate k_ATP_, in all cases. We consider instead three cases for the dimerization reaction. In our baseline model, the conversion of M into D also occurred at the same rate in the cytoplasm, at the membrane, and at the pole. We have set this rate such that the M and D states are on average comparable in steady state, as suggested by our experiments. We also considered the case where dimerization at the membrane occurs twofold more often at the membrane, and a third case where polar localization (through MinJ interaction) strongly stimulates dimerization, so that the M to D transition occurs at a rate k_pole_ >>k_ATP_ at poles. When considering polar stimulation, dimerization occurs at a rate k_ATP_/10 in the cytosol; otherwise, rates of dimerization and monomerization in the cytoplasm are equal. The diffusion of monomeric MinD was set to 0.0033 in simulation units (corresponding to approximately 2 µm^2^/s, see below), where the hydrolysis rate k_ATP_ was set to 10^–5^ (corresponding to 1/s, which is of the same order as used in previous modeling studies; Huang et al., 2003). When considering polar stimulation, we set k_pole_ such that dimerization at the pole occurs with unit probability during a timestep (we have verified though that the results are qualitatively similar down to at least a value of k_pole_ equal to 100 k_ATP_). More details on the parameters used in the model are given in the caption of Figure 8 and Supplementary file 1.

To simulate the monomer (K16A) and dimer (D40A) mutants, we used another version of the same model, in which all particles were of type M or D, respectively, and the rate of interconversion between the two was set to 0.

Simulation units may be mapped to physical units by noting that one space and time unit in simulations can be made to correspond to 0.08 µm and 10 µs, respectively. Simulations were run typically for 10^7^ time steps. Snapshots shown are for systems which have reached equilibrium, so representing steady-state behavior.

## Figures and Tables

**Appendix 1—figure 1.**
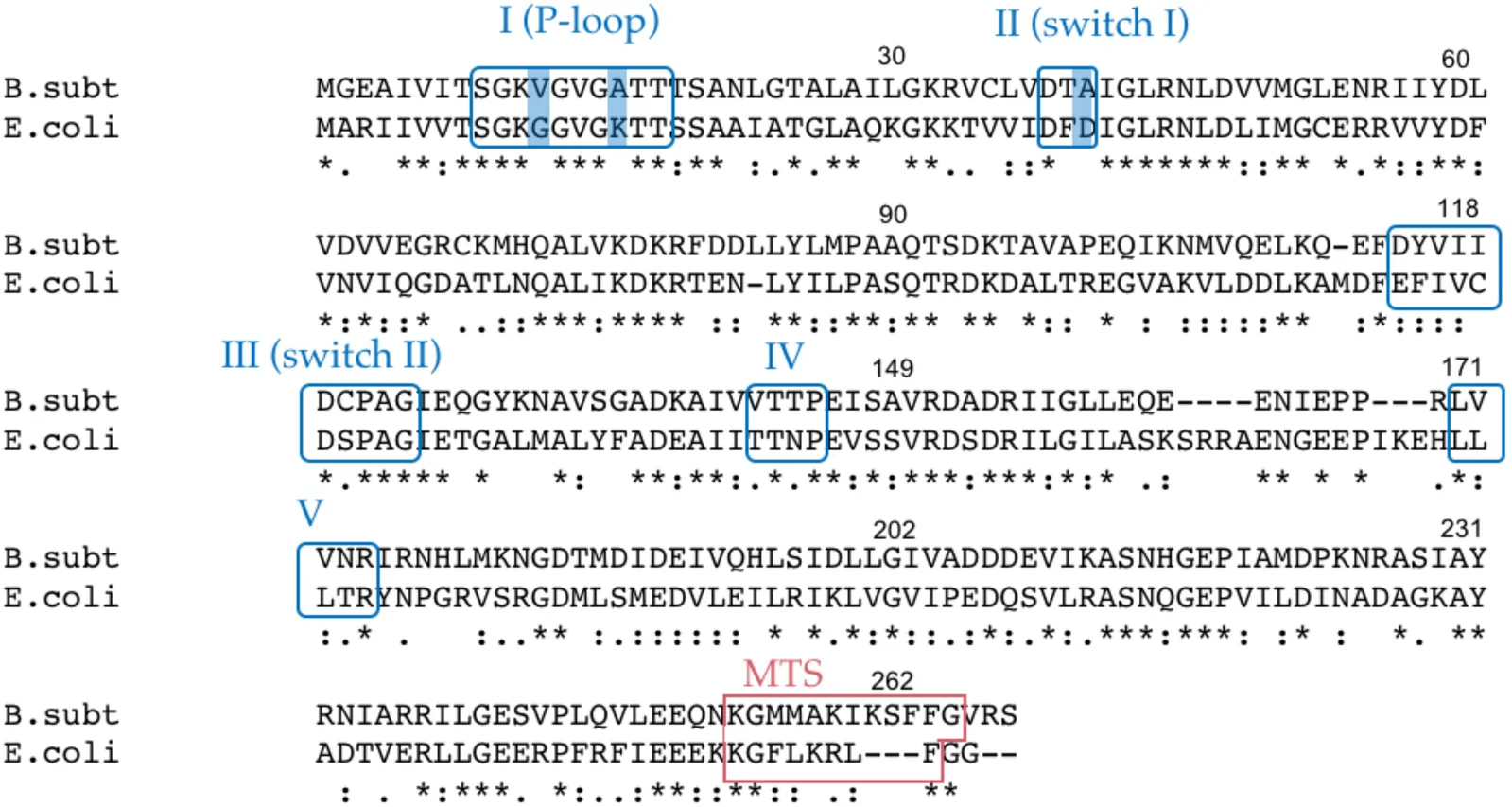
Amino acid sequence alignment between *E. coli* and *B. subtilis* MinD proteins. The *E. coli* and *B. subtilis* MinD proteins share 44% identical residues. The five conserved motifs of the ATPase protein family are framed in blue. The exchanged conserved amino acids are highlighted in blue (G12V, K16A, D40A). The C-terminal membrane-targeting amphipathic helices are framed in red (MTS).

**Appendix 1—figure 2.**
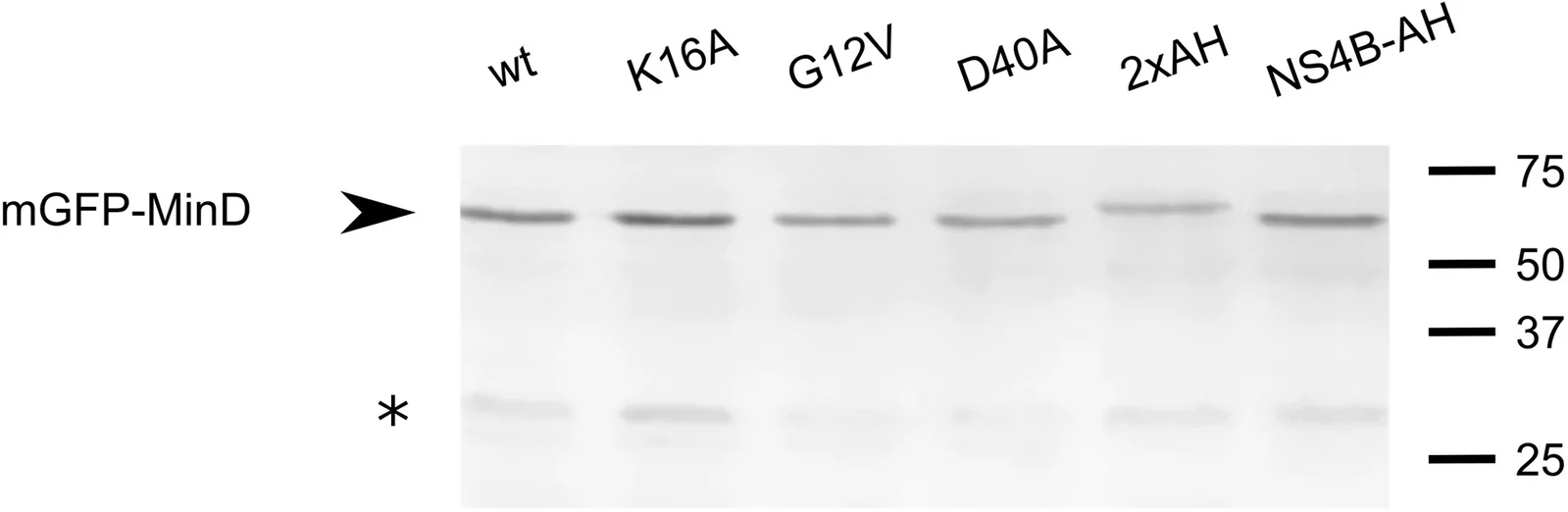
Western blot analysis of GFP-MinD variants. Western blot analysis of strains expressing different mGFP-MinD protein fusions. Cells were grown in LB medium at 37°C until early exponential phase, followed by induction for three doubling times with 0.1% xylose. Anti-GFP primary antibody was used to detect the different fusion proteins. Strains used: LB305 (wt), LB306 (K16A), LB307 (G12V), LB308 (D40A), LB507 (2xAH), LB508 (NS4B-AH). Black arrowhead indicates full-length mGFP-MinD (~57.7 kDa), the asterisk indicates a nonspecific band. Appendix 1—figure 2—source data 1.Original Western blot Appendix 1—figure 2. Appendix 1—figure 2—source data 2.Original Western blot with markings Appendix 1—figure 2.

**Appendix 1—figure 3.**
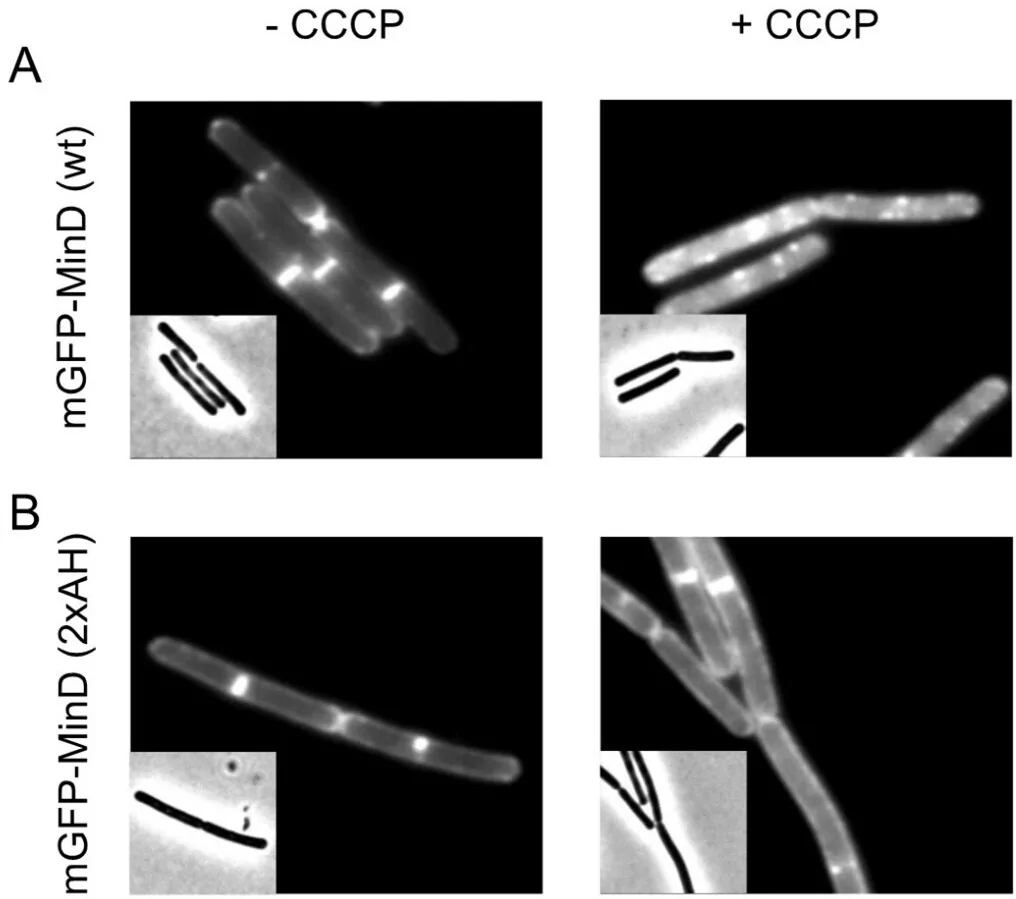
Effect of membrane potential dissipation on membrane binding. Fluorescence images of cells expressing mGFP-MinD variants with the wild-type amphipathic helix (wt) (**A**) and the strong (2xAH) membrane binding amphipathic helix (**B**). Corresponding phase contrast images are shown in the insets. The membrane potential was dissipated by the proton-ionophore carbonyl cyanide *m*-chlorophenylhydrazone (CCCP) (100 μM for 10 min). mGFP-MinD fusions were expressed from the ectopic *amyE* locus using a xylose-inducible promoter and a Δ*minD* background. Strains used: (**A**) LB249, (**B**) LB507.

## Data Availability

All strains constructed during this study are available upon request, and all data analyzed during this study are included in the manuscript and supporting files. Numerical source data underlying the graphs of the manuscript is provided in the online version, and software and mathematical algorithms are available at https://git.ecdf.ed.ac.uk/dmarendu/mind_modelling and uploaded as Source code 1.

## Additional files

Supplementary file 1. Table with parameter values in simulation and physical units.

MDAR checklist

Source code 1. Basic codes needed to model MinD gradient patterns in *Bacillus subtilis*.

## References

[bib1] Adams DW, Wu LJ, Errington J (2015). Nucleoid occlusion protein Noc recruits DNA to the bacterial cell membrane. The EMBO Journal.

[bib2] Allen MP, Tildesley DJ (1987). Computer Simulations of Liquids.

[bib3] Barbe V, Cruveiller S, Kunst F, Lenoble P, Meurice G, Sekowska A, Vallenet D, Wang T, Moszer I, Médigue C, Danchin A (2009). From a consortium sequence to a unified sequence: the *Bacillus subtilis* 168 reference genome a decade later. Microbiology.

[bib4] Bi E, Lutkenhaus J (1993). Cell division inhibitors SulA and MinCD prevent formation of the FtsZ ring. Journal of Bacteriology.

[bib5] Binder K, Heermann DW (2002). Monte Carlo Simulation in Statistical Physics: An Introduction.

[bib6] Bramkamp M, Emmins R, Weston L, Donovan C, Daniel RA, Errington J (2008). A novel component of the division-site selection system of *Bacillus subtilis* and a new mode of action for the division inhibitor MinCD. Molecular Microbiology.

[bib7] Conti J, Viola MG, Camberg JL (2015). The bacterial cell division regulators MinD and MinC form polymers in the presence of nucleotide. FEBS Letters.

[bib8] Cordell SC, Anderson RE, Löwe J (2001). Crystal structure of the bacterial cell division inhibitor MinC. The EMBO Journal.

[bib9] de Boer PA, Crossley RE, Rothfield LI (1989). A division inhibitor and a topological specificity factor coded for by the minicell locus determine proper placement of the division septum in *E. coli*. Cell.

[bib10] de Boer PA, Crossley RE, Hand AR, Rothfield LI (1991). The MinD protein is a membrane ATPase required for the correct placement of the *Escherichia coli* division site. The EMBO Journal.

[bib11] de Boer PA, Crossley RE, Rothfield LI (1992). Roles of MinC and MinD in the site-specific septation block mediated by the MinCDE system of *Escherichia coli*. Journal of Bacteriology.

[bib12] Deeng J, Chan KY, van der Sluis EO, Berninghausen O, Han W, Gumbart J, Schulten K, Beatrix B, Beckmann R (2016). Dynamic behavior of trigger factor on the ribosome. Journal of Molecular Biology.

[bib13] Di Ventura B, Sourjik V (2011). Self-organized partitioning of dynamically localized proteins in bacterial cell division. Molecular Systems Biology.

[bib14] Feddersen H, Würthner L, Frey E, Bramkamp M (2021). Dynamics of the *Bacillus subtilis* min system. mBio.

[bib15] Feddersen H, Bramkamp M (2024). Mechanistic Insights into MinD Regulation and Pattern Formation in *Bacillus subtilis*. bioRxiv.

[bib16] Fort P, Errington J (1985). Nucleotide sequence and complementation analysis of a polycistronic sporulation operon, spoVA, in *Bacillus subtilis*. Journal of General Microbiology.

[bib17] Ghosal D, Trambaiolo D, Amos LA, Löwe J (2014). MinCD cell division proteins form alternating copolymeric cytomotive filaments. Nature Communications.

[bib18] Glock P, Brauns F, Halatek J, Frey E, Schwille P (2019). Design of biochemical pattern forming systems from minimal motifs. eLife.

[bib19] Gouttenoire J, Montserret R, Kennel A, Penin F, Moradpour D (2009). An amphipathic alpha-helix at the C terminus of hepatitis C virus nonstructural protein 4B mediates membrane association. Journal of Virology.

[bib20] Gregory JA, Becker EC, Pogliano K (2008). *Bacillus subtilis* MinC destabilizes FtsZ-rings at new cell poles and contributes to the timing of cell division. Genes & Development.

[bib21] Hamoen LW, Smits WK, de Jong A, Holsappel S, Kuipers OP (2002). Improving the predictive value of the competence transcription factor (ComK) binding site in *Bacillus subtilis* using a genomic approach. Nucleic Acids Research.

[bib22] Hayashi I, Oyama T, Morikawa K (2001). Structural and functional studies of MinD ATPase: implications for the molecular recognition of the bacterial cell division apparatus. The EMBO Journal.

[bib23] Heermann T, Ramm B, Glaser S, Schwille P (2020). Local self-enhancement of MinD membrane binding in min protein pattern formation. Journal of Molecular Biology.

[bib24] Howard M, Rutenberg AD, de Vet S (2001). Dynamic compartmentalization of bacteria: accurate division in *E. coli*. Physical Review Letters.

[bib25] Howard M (2004). A mechanism for polar protein localization in bacteria. Journal of Molecular Biology.

[bib26] Hu Z, Lutkenhaus J (1999). Topological regulation of cell division in *Escherichia coli* involves rapid pole to pole oscillation of the division inhibitor MinC under the control of MinD and MinE. Molecular Microbiology.

[bib27] Hu Z, Lutkenhaus J (2000). Analysis of MinC reveals two independent domains involved in interaction with MinD and FtsZ. Journal of Bacteriology.

[bib28] Hu Z, Lutkenhaus J (2001). Topological regulation of cell division in *E. coli*. spatiotemporal oscillation of MinD requires stimulation of its ATPase by MinE and phospholipid. Molecular Cell.

[bib29] Hu Z, Gogol EP, Lutkenhaus J (2002). Dynamic assembly of MinD on phospholipid vesicles regulated by ATP and MinE. PNAS.

[bib30] Hu Z, Lutkenhaus J (2003). A conserved sequence at the C-terminus of MinD is required for binding to the membrane and targeting MinC to the septum. Molecular Microbiology.

[bib31] Hu Z, Saez C, Lutkenhaus J (2003). Recruitment of MinC, an inhibitor of Z-ring formation, to the membrane in *Escherichia coli*: role of MinD and MinE. Journal of Bacteriology.

[bib32] Huang KC, Meir Y, Wingreen NS (2003). Dynamic structures in *Escherichia coli*: spontaneous formation of MinE rings and MinD polar zones. PNAS.

[bib33] Huang H, Wang P, Bian L, Osawa M, Erickson HP, Chen Y (2018). The cell division protein MinD from *Pseudomonas aeruginosa* dominates the assembly of the MinC-MinD copolymers. The Journal of Biological Chemistry.

[bib34] Ishikawa K, Matsuoka S, Hara H, Matsumoto K (2017). Septal membrane localization by C-terminal amphipathic α-helices of MinD in *Bacillus subtilis* mutant cells lacking MinJ or DivIVA. Genes & Genetic Systems.

[bib35] Karoui ME, Errington J (2001). Isolation and characterization of topological specificity mutants of minD in *Bacillus subtilis*. Molecular Microbiology.

[bib36] Kruse K (2002). A dynamic model for determining the middle of *Escherichia coli*. Biophysical Journal.

[bib37] Kumar M, Mommer MS, Sourjik V (2010). Mobility of cytoplasmic, membrane, and DNA-binding proteins in *Escherichia coli*. Biophysical Journal.

[bib38] Lackner LL, Raskin DM, de Boer PAJ (2003). ATP-dependent interactions between *Escherichia coli* Min proteins and the phospholipid membrane in vitro. Journal of Bacteriology.

[bib39] Landgraf D, Okumus B, Chien P, Baker TA, Paulsson J (2012). Segregation of molecules at cell division reveals native protein localization. Nature Methods.

[bib40] Leipe DD, Wolf YI, Koonin EV, Aravind L (2002). Classification and evolution of P-loop GTPases and related ATPases. Journal of Molecular Biology.

[bib41] Lenarcic R, Halbedel S, Visser L, Shaw M, Wu LJ, Errington J, Marenduzzo D, Hamoen LW (2009). Localisation of DivIVA by targeting to negatively curved membranes. The EMBO Journal.

[bib42] Leonard TA, Butler PJ, Löwe J (2005). Bacterial chromosome segregation: structure and DNA binding of the Soj dimer--a conserved biological switch. The EMBO Journal.

[bib43] Levin PA, Shim JJ, Grossman AD (1998). Effect of minCD on FtsZ ring position and polar septation in *Bacillus subtilis*. Journal of Bacteriology.

[bib44] Lewis PJ, Marston AL (1999). GFP vectors for controlled expression and dual labelling of protein fusions in *Bacillus subtilis*. Gene.

[bib45] Loose M, Fischer-Friedrich E, Ries J, Kruse K, Schwille P (2008). Spatial regulators for bacterial cell division self-organize into surface waves in vitro. Science.

[bib46] MacCready JS, Hakim P, Young EJ, Hu L, Liu J, Osteryoung KW, Vecchiarelli AG, Ducat DC (2018). Protein gradients on the nucleoid position the carbon-fixing organelles of cyanobacteria. eLife.

[bib47] Marston AL, Thomaides HB, Edwards DH, Sharpe ME, Errington J (1998). Polar localization of the MinD protein of *Bacillus subtilis* and its role in selection of the mid-cell division site. Genes & Development.

[bib48] Marston AL, Errington J (1999). Selection of the midcell division site in *Bacillus subtilis* through MinD-dependent polar localization and activation of MinC. Molecular Microbiology.

[bib49] Meinhardt H, de Boer PA (2001). Pattern formation in *Escherichia coli*: a model for the pole-to-pole oscillations of Min proteins and the localization of the division site. PNAS.

[bib50] Mika JT, Thompson AJ, Dent MR, Brooks NJ, Michiels J, Hofkens J, Kuimova MK (2016). Measuring the viscosity of the *Escherichia coli* plasma membrane using molecular rotors. Biophysical Journal.

[bib51] Morimoto T, Loh PC, Hirai T, Asai K, Kobayashi K, Moriya S, Ogasawara N (2002). Six GTP-binding proteins of the Era/Obg family are essential for cell growth in *Bacillus subtilis*. Microbiology.

[bib52] Mullineaux CW, Nenninger A, Ray N, Robinson C (2006). Diffusion of green fluorescent protein in three cell environments in *Escherichia coli*. Journal of Bacteriology.

[bib53] Nordholt N, van Heerden J, Kort R, Bruggeman FJ (2017). Effects of growth rate and promoter activity on single-cell protein expression. Scientific Reports.

[bib54] Park KT, Wu W, Lovell S, Lutkenhaus J (2012). Mechanism of the asymmetric activation of the MinD ATPase by MinE. Molecular Microbiology.

[bib55] Park KT, Du S, Lutkenhaus J (2015). MinC/MinD copolymers are not required for Min function. Molecular Microbiology.

[bib56] Park KT, Villar MT, Artigues A, Lutkenhaus J (2017). MinE conformational dynamics regulate membrane binding, MinD interaction, and Min oscillation. PNAS.

[bib57] Patrick JE, Kearns DB (2008). MinJ (YvjD) is a topological determinant of cell division in *Bacillus subtilis*. Molecular Microbiology.

[bib58] Ptacin JL, Lee SF, Garner EC, Toro E, Eckart M, Comolli LR, Moerner WE, Shapiro L (2010). A spindle-like apparatus guides bacterial chromosome segregation. Nature Cell Biology.

[bib59] Ramamurthi KS, Losick R (2009). Negative membrane curvature as a cue for subcellular localization of a bacterial protein. PNAS.

[bib60] Ramm B, Heermann T, Schwille P (2019). The *E. coli* MinCDE system in the regulation of protein patterns and gradients. Cellular and Molecular Life Sciences.

[bib61] Raskin DM, de Boer PA (1999). Rapid pole-to-pole oscillation of a protein required for directing division to the middle of *Escherichia coli*. PNAS.

[bib62] Rowlett VW, Margolin W (2013). The bacterial Min system. Current Biology.

[bib63] Sanz E, Marenduzzo D (2010). Dynamic monte carlo versus brownian dynamics: a comparison for self-diffusion and crystallization in colloidal fluids. The Journal of Chemical Physics.

[bib64] Story RM, Steitz TA (1992). Structure of the recA protein-ADP complex. Nature.

[bib65] Strahl H, Hamoen LW (2010). Membrane potential is important for bacterial cell division. PNAS.

[bib66] Suefuji K, Valluzzi R, RayChaudhuri D (2002). Dynamic assembly of MinD into filament bundles modulated by ATP, phospholipids, and MinE. PNAS.

[bib67] Surovtsev IV, Lim HC, Jacobs-Wagner C (2016). The slow mobility of the para partitioning protein underlies its steady-state patterning in caulobacter. Biophysical Journal.

[bib68] Syvertsson S, Wang B, Staal J, Gao Y, Kort R, Hamoen LW (2021). Different resource allocation in a *Bacillus subtilis* population displaying bimodal motility. Journal of Bacteriology.

[bib69] Szeto TH, Rowland SL, Habrukowich CL, King GF (2003). The MinD membrane targeting sequence is a transplantable lipid-binding helix. The Journal of Biological Chemistry.

[bib70] van Baarle S, Bramkamp M (2010). The MinCDJ system in *Bacillus subtilis* prevents minicell formation by promoting divisome disassembly. PLOS ONE.

[bib71] Veiga H, Jousselin A, Schaper S, Saraiva BM, Marques LB, Reed P, Wilton J, Pereira PM, Filipe SR, Pinho MG (2023). Cell division protein FtsK coordinates bacterial chromosome segregation and daughter cell separation in *Staphylococcus aureus*. The EMBO Journal.

[bib72] Vischer NOE, Verheul J, Postma M, van den Berg van Saparoea B, Galli E, Natale P, Gerdes K, Luirink J, Vollmer W, Vicente M, den Blaauwen T (2015). Cell age dependent concentration of *Escherichia coli* divisome proteins analyzed with ImageJ and ObjectJ. Frontiers in Microbiology.

[bib73] Wang N, Zhang T, Du S, Zhou Y, Chen Y (2022). How Do MinC-D copolymers act on Z-Ring localization regulation? a new model of *Bacillus subtilis* min system. Frontiers in Microbiology.

[bib74] Whitelam S, Geissler PL (2007). Avoiding unphysical kinetic traps in Monte Carlo simulations of strongly attractive particles. The Journal of Chemical Physics.

[bib75] Wu W, Park KT, Holyoak T, Lutkenhaus J (2011). Determination of the structure of the MinD-ATP complex reveals the orientation of MinD on the membrane and the relative location of the binding sites for MinE and MinC. Molecular Microbiology.

[bib76] Würthner L, Brauns F, Pawlik G, Halatek J, Kerssemakers J, Dekker C, Frey E (2022). Bridging scales in a multiscale pattern-forming system. PNAS.

