# Supplementary material for: Membrane affinity difference between MinD monomer and dimer is not crucial for MinD gradient formation in Bacillus subtilis
Source: eLife. 2026 Sep 2;13:RP101520. doi: 10.7554/eLife.101520 (PMC13537761; doi:10.7554/eLife.101520)
Supplement: Supplementary file 2 [file elife-101520-supp1.docx]

**Supplementary file 1**

**Table with parameter values in simulation and physical units**

Mapping has been made considering one spatial and temporal simulation units correspond to $0.08 \mu m$ and $10 \mu s$ respectively; different assumptions will lead to different mapping which can be calculated by scaling the values shown in this Table according to the physical units of the corresponding quantity. As described in the Methods, we have called $D_{m}, D_{d}$ the diffusion coefficient of the monomer and the dimer respectively; whereas $p_{MD}^{pole}, p_{MD}^{mem}, p_{MD}$ denote the rates of monomer to dimer conversion at the pole, membrane and in the bulk respectively, and $k_{m}=k_{ATP}$ denotes the rate of dimer to monomer conversion. Other parameters are: (i) $\epsilon_{M}, \epsilon_{D}, \epsilon_{C}$, which are respectively the interaction energy between membrane and monomer, between membrane and dimer, and between spherocylinder cap and dimer; (ii) $R_{cyl}$ and $h_{cyl}$, which denote the radius and height of the spherocylinder that models the bacterium.

| Parameter | Value (Simulation Units) | Value (Physical Units) |
| --- | --- | --- |
| $D_{m}$ | $0.0033$ | $\simeq2.1 \mu m^{2}/s$ |
| $D_{d}$ | $0.0017$ | $\simeq1.1 \mu m^{2}/s$ |
| $p_{MD}^{pole}=k_{pole}$ | $1$ | ${10}^{5}s^{-1}$ |
| $p_{MD}^{mem}$ | $2\times{10}^{-6}$ | $0.2 s^{-1}$ |
| $p_{MD}$ | ${10}^{-6}$ | $0.1 s^{-1}$ |
| $k_{m}=k_{ATP}$ | ${10}^{-5}$ | $1 s^{-1}$ |
| $\epsilon_{M}$ | $1$ | $k_{B}T\simeq4.1\times{10}^{-21}J$ |
| $\epsilon_{D}$ | $1.1$ | $1.1 k_{B}T$ |
| $\epsilon_{C}$ | $0.7$ | ${0.7 k}_{B}T$ |
| $R_{cyl}$ | $10$ | $0.8 \mu m$ |
| $h_{cyl}$ | $80$ | $6.4 \mu m$ |
