# Supplementary material for: Membrane affinity difference between MinD monomer and dimer is not crucial for MinD gradient formation in Bacillus subtilis
Source: eLife. 2026 Sep 2;13:RP101520. doi: 10.7554/eLife.101520 (PMC13537761; doi:10.7554/eLife.101520)
Supplement: Peer review history

# Editor report: eLife Assessment

**DOI:** 10.7554/eLife.101520.4.sa0

**Authors:** Petra Anne Levin

**Affiliations:** Washington University in St. Louis United States

**Keywords:** Compelling, Important

In the gram-positive model organism *Bacillus subtilis*, the membrane associated ParA family member MinD, concentrates the division inhibitor MinC at cell poles where it prevents aberrant division events. This **important** study presents **compelling** data suggesting that polar localization of MinCD is largely due to differences in diffusion rates between monomeric and dimeric MinD. This finding is exciting as it negates the necessity for a third, localization determinant, in this system as has been proposed by previous investigations.

# Referee report: Reviewer #1 (Public review)

**DOI:** 10.7554/eLife.101520.4.sa1

The authors used fluorescence microscopy, image analysis, and mathematical modeling to study the effects of membrane affinity and diffusion rates of MinD monomer and dimer states on MinD gradient formation in *B. subtilis*. To test these effects, the authors experimentally examined MinD mutants that lock the protein in specific states, including Apo monomer (K16A), ATP-bound monomer (G12V) and ATP-bound dimer (D40A, hydrolysis defective), and compared to wild-type MinD. Overall, the experimental results support the conclusions that reversible membrane binding of MinD is critical for the formation of the MinD gradient, but the binding affinities between monomers and dimers are similar.

The modeling part is a new attempt to use the Monte Carlo method to test the conditions for the formation of the MinD gradient in *B. subtilis*. The modeling results provide good support for the observations and find that the MinD gradient is sensitive to different diffusion rates between monomers and dimers. This simulation is based on several assumptions and predictions, which raises new questions that need to be addressed experimentally in the future.

# Referee report: Reviewer #3 (Public review)

**DOI:** 10.7554/eLife.101520.4.sa2

This important study by Bohorquez et al examines the determinants necessary for concentrating the spatial modulator of cell division, MinD, at the future site of division and the cell poles. Proper localization of MinD is necessary to bring the division inhibitor, MinC, in proximity to the cell membrane and cell poles where it prevents aberrant assembly of the division machinery. In contrast to *E. coli*, in which MinD oscillates from pole-to-pole courtesy of a third protein MinE, how MinD localization is achieved in *B. subtilis*-which does not encode a MinE analog-has remained largely a mystery. The authors present compelling data indicating that MinD dimerization is dispensable for membrane localization but required for concentration at the cell poles. Dimerization is also important for interactions between MinD and MinC, leading to the formation of large protein complexes. Computational modeling, specifically a Monte Carlo simulation, supports a model in which differences in diffusion rates between MinD monomers and dimers lead to concentration of MinD at cell poles. Once there, interaction with MinC increases the size of the complex, further reinforcing diffusion differences. Notably, interactions with MinJ-which has previously been implicated in MinCD localization, are dispensable for concentrating MinD at cell poles although MinJ may help stabilize the MinCD complex at those locations.

[Editor's note: The editors and reviewers have no further comments and encourage the authors to proceed with a Version of Record.]

# Author comment: Author response

**DOI:** 10.7554/eLife.101520.4.sa3

**Authors:** Laura C Bohorquez, Henrik Strahl, Davide Marenduzzo, Martin J Thiele, Frank Burmann, Leendert Hamoen

**Affiliations:** FIND | Campus Biotech Geneva Switzerland; Newcastle University Newcastle upon Tyne United Kingdom; University of Edinburgh Edinburgh United Kingdom; Carl Zeiss Meditec AG Jena Germany; University of Oxford Oxford United Kingdom; University of Amsterdam Amsterdam Netherlands

The following is the authors’ response to the previous reviews

**Public Review:**

**Reviewer #1 (Public review):**

The authors used fluorescence microscopy, image analysis, and mathematical modeling to study the effects of membrane affinity and diffusion rates of MinD monomer and dimer states on MinD gradient formation in *B. subtilis.* To test these effects, the authors experimentally examined MinD mutants that lock the protein in specific states, including Apo monomer (K16A), ATP-bound monomer (G12V) and ATP-bound dimer (D40A, hydrolysis defective), and compared to wild-type MinD. Overall, the experimental results support the conclusions that reversible membrane binding of MinD is critical for the formation of the MinD gradient, but the binding affinities between monomers and dimers are similar.

The modeling part is a new attempt to use the Monte Carlo method to test the conditions for the formation of the MinD gradient in *B. subtilis*. The modeling results provide good support for the observations and find that the MinD gradient is sensitive to different diffusion rates between monomers and dimers. This simulation is based on several assumptions and predictions, which raises new questions that need to be addressed experimentally in the future.

**Reviewer #3 (Public review):**

This important study by Bohorquez et al examines the determinants necessary for concentrating the spatial modulator of cell division, MinD, at the future site of division and the cell poles. Proper localization of MinD is necessary to bring the division inhibitor, MinC, in proximity to the cell membrane and cell poles where it prevents aberrant assembly of the division machinery. In contrast to *E. coli*, in which MinD 50 oscillates from pole-to-pole courtesy of a third protein MinE, how MinD localization is achieved in *B. subtilis*-which does not encode a MinE analog-has remained largely a mystery. The authors present 52 compelling data indicating that MinD dimerization is dispensable for membrane localization but required 53 for concentration at the cell poles. Dimerization is also important for interactions between MinD and MinC, 54 leading to the formation of large protein complexes. Computational modeling, specifically a Monte Carlo 55 simulation, supports a model in which differences in diffusion rates between MinD monomers and dimers 56 lead to concentration of MinD at cell poles. Once there, interaction with MinC increases the size of the 57 complex, further reinforcing diffusion differences. Notably, interactions with MinJ-which has previously 58 been implicated in MinCD localization, are dispensable for concentrating MinD at cell poles although MinJ may help stabilize the MinCD complex at those locations.

Comments on revisions:

I believe the authors put respectable effort into revisions and addressing reviewer comments, particularly 64 those that focused on the strengths of the original conclusions. The language in the current version of the manuscript is more precise and the overall product is stronger.

We are happy to learn that the reviewer considers our manuscript ready for publication.

**Recommendations for the authors:**

**Reviewer #1 (Recommendations for the authors):**

The author has adequately answered the questions that were raised in my previous comments. There are only few minor revisions needed for improvement.

Line 48−49: 'These proteins ensure that cell division occurs at midcell and not close to nascent division sites or cell poles'

delete 'nascent division site'

This has now been corrected as suggested.

Line 64−65: 'MinC inhibits polymerization of FtsZ by direct protein-protein interactions and needs to bind to the Walker A-type ATPase MinD for its recruitment to septa or the polar regions of the cell'

delete 'septa or', because MinD recruits MinC to the cell poles to block polar division, not septal formation.

This has now been corrected as suggested.

Supplemental information:

Some parameters in Table S1 are missing definitions. If these parameters relate to terms described in the "Methods" section, please add the corresponding parameter symbols after the terms.

We would like to thank the reviewer for pointing this out. We have improved Table S1 and corrected the related parameters in the Methods section (lines 605-619).
